# Emerging Technology-Supported Intercultural Learning for Cognitive, Attitudinal, and Behavioral Outcomes: A Systematic Review and Three-Level Meta-Analysis

**DOI:** 10.3390/jintelligence14100265

**Published:** 2026-10-16

**Authors:** Yuteng Wang, Junjie Gavin Wu, Danyang Zhang

**Affiliations:** 1 Faculty of Applied Sciences, Macao Polytechnic University, R. de Luís Gonzaga Gomes, Macao SAR 999078, China; 2 Faculty of Languages and Translation, Macao Polytechnic University, R. de Luís Gonzaga Gomes, Macao SAR 999078, China; 3 School of Foreign Languages, Shenzhen University, 3688 Nanhai Avenue, Nanshan District, Shenzhen 518060, China

**Keywords:** emerging technology, intercultural learning, three-level meta-analysis, artificial intelligence, augmented reality, virtual reality

## Abstract

Emerging technologies are increasingly used to support intercultural learning, yet evidence on the distinct effects of artificial intelligence (AI), augmented reality (AR), and virtual reality (VR) remains limited. This systematic review and three-level meta-analysis synthesized 17 studies published from 2020 to July 2026 (77 effect sizes) to examine cognitive, attitudinal, and behavioral outcomes. The overall effect was large and significant (Hedges’ g = 0.965, 95% CI [0.580, 1.350], *p* < .001), although heterogeneity was substantial. AI-supported interventions also showed a significant positive pooled effect (g = 1.150, 95% CI [0.744, 1.555], *p* < .001). AR showed a significantly larger estimated effect than VR (*β* = −1.481 for VR relative to AR, *p* = .017), but this difference was not robust to excluding one AR study. Academic level (*F* (1, 75) = 17.27, *p* < .001) and sample size (*F* (2, 74) = 3.873, *p* = .026) were significant moderators, with larger effects in K–12 and larger-sample studies. However, these exploratory comparisons involved uneven technology distributions and only two K–12 studies. Outcome domain did not significantly moderate effects. Sensitivity analyses supported the stability of the overall pooled effect. The findings indicate that emerging technologies can improve intercultural learning, while better-balanced comparative studies are needed to identify which technological approaches are most effective.

## 1. Introduction

As globalization, international mobility, and digital communication increasingly require learners to engage with people and practices from different cultural backgrounds, intercultural learning has become an important component of contemporary education (Al-Afifi et al., 2026). In general, intercultural learning refers to the process in which learners understand cultural differences and develop the ability to communicate appropriately with people from other cultural backgrounds. Intercultural learning extends beyond the acquisition of cultural knowledge to encompass intercultural awareness, openness, perspective-taking, and critical reflection (Al-Afifi et al., 2026). In the past five years, the rapid development of emerging technologies has created new possibilities for intercultural learning. Artificial intelligence (AI) makes intercultural learning more interactive and personalized because conversational agents can answer learners’ cultural questions, provide examples from different perspectives and give feedback during learning (L. Sun, 2025). Extended reality (XR) makes intercultural learning more situated and experiential because augmented reality (AR) and virtual reality (VR) environments can present cultural practices, customs and communicative norms in simulated contexts (J. G. Wu et al., 2022; D. Zhang et al., 2020).

Fukkink et al. (2024) conducted a systematic review of Collaborative Online International Learning and virtual exchange studies. They classified intercultural learning outcomes into affective, behavioral, and cognitive domains. The present study adopts this outcome-based framework. It uses the term attitudinal for the affective domain and classifies the extracted outcomes as attitudinal, behavioral, or cognitive. Attitudinal outcomes encompass learners’ openness, empathy, intercultural sensitivity, and willingness to engage with cultural differences. Behavioral outcomes encompass participation, interaction skills, communicative actions, and appropriate responses in intercultural situations. Cognitive outcomes encompass cultural knowledge, cultural understanding, and the interpretation of cultural differences. This framework provides an operational basis for classifying outcomes in the included studies and for assessing how previous syntheses treated different outcome domains; it is not intended to define the broader construct of intercultural competence. These three outcome domains can also be linked to cultural intelligence (CQ). Cognitive outcomes involve understanding and interpreting cultural knowledge. Attitudinal outcomes, including openness and empathy, are conceptually related to, but not equivalent to, motivational CQ, which emphasizes motivation and self-efficacy in intercultural interactions (Ang et al., 2007). Metacognitive CQ is also relevant because it involves reflecting on and adjusting one’s understanding of unfamiliar cultural situations (Ang et al., 2007). Although these outcomes are not the same as CQ, they are related to the broader processes of understanding and adapting to cultural differences (Sternberg et al., 2022).

Previous syntheses provide an important basis for understanding technology-supported intercultural learning. Shadiev and Dang (2022) provided an earlier systematic review of studies published between 2010 and 2021. Rather than estimating pooled intervention effects, their review described how different technologies were used to support intercultural contact, information exchange, collaboration, and reflection across learning contexts. X. Zhang and Zhou (2023) subsequently conducted a quantitative meta-analysis of 56 studies published between 2000 and 2021. They examined overall intercultural competence and its affective, cognitive, and behavioral dimensions, while classifying technology-supported interventions into computer simulation, blended learning environments, digital materials and tools, and computer-mediated communication. Their moderator analyses identified intervention type and measurement instrument type as significant sources of variation. However, these technology categories remained relatively broad, making it difficult to distinguish the effects of specific emerging technologies. More recently, Shadiev et al. (2026) synthesized 30 studies published between 2017 and 2025 and examined instrument type, academic level, intervention technology, intervention type, intervention duration, publication year, and sample size as moderators. Their technology classification included online course systems, multimedia resources, social media, VR/AR, and online meeting tools, with AI language tools additionally examined in the within-subject analyses. AR and VR were not analyzed separately. Xiao et al. (2026) further analyzed 21 studies published between 2020 and 2025 using a three-level meta-analytic model. They classified learning outcomes as knowledge, skills, and attitudes and examined course type, intervention type, intervention duration, and cultural background. However, technologies were primarily grouped into online communication and immersive experiences, with AR and VR included within the broader immersive category. Taken together, these syntheses provide increasingly detailed evidence on the effectiveness and moderators of technology-supported intercultural learning, but they offer limited evidence on the distinct effects of specific emerging technologies, particularly AI, AR, and VR.

This study extends previous syntheses in both recency and analytical granularity (Table 1). It includes 17 studies published from 1 January 2020 to 31 July 2026, and focuses specifically on AI-, AR-, and VR-supported intercultural learning. Eight of the included studies were published in 2026. None of the 17 studies overlapped with X. Zhang and Zhou (2023), while three were also included in Xiao et al. (2026). More importantly, this study distinguishes AR from VR and analyzes AI-supported interventions separately rather than combining specific technologies within broader categories such as immersive experiences. For outcome classification, this study adapts the cognitive, affective, and behavioral framework of Fukkink et al. (2024), with the affective domain further operationalized as attitudinal. Outcomes are classified into cognitive, attitudinal, and behavioral domains based on their underlying constructs, item content, and scoring criteria. This classification allows the study to test whether the effects of emerging technology-supported intercultural learning differ across cognitive, attitudinal, and behavioral domains. Together with the finer technology classification, it also enables a between-study subgroup comparison of AR- and VR-based interventions and a separate estimation of the pooled effect of AI-supported interventions. Thus, the incremental contribution of this study lies in its technology-specific analysis rather than the outcome classification itself.

Academic level, sample size, and measurement instrument type may explain different sources of variation in the reported effects. Academic level represents the developmental and educational context of intercultural learning. Previous meta-analytic evidence showed that grade level moderated the effects of intercultural competence interventions (X. Zhang & Zhou, 2019). Academic level may be especially relevant when emerging technologies are used. Research on mobile AR has identified several learning affordances, including authenticity, interactivity, immersion, and engagement. It has also emphasized the importance of theoretical grounding and effective task design (D. Zhang et al., 2020; D. Zhang & Wu, 2026). Learners at different academic levels may therefore respond differently to these features. Sample size and measurement instrument type represent methodological characteristics that may contribute to variation in reported effects. Estimates from smaller studies are generally less precise. Smaller studies may also be more affected by sampling variation and selective reporting. These conditions can produce small-study effects (Nakagawa et al., 2022; Sterne et al., 2000). X. Zhang and Zhou (2023) did not find a significant moderating effect of sample size. However, they found that studies using self-developed measures reported larger effects than those using validated scales, possibly because study-specific measures were more closely aligned with intervention content and more sensitive to intervention-related changes. Their study examined information and digital technologies using broad categories. Re-examining both sample size and measurement instrument type after separating AI, AR, and VR may show whether these methodological characteristics contribute to variation in the estimated effects.

Based on these gaps, this study aims to provide clearer evidence on how emerging technologies support intercultural learning. First, the analysis examines the overall effect of emerging technology-supported learning on learners’ intercultural outcomes. Then, the study compares the effects across attitudinal, behavioral, and cognitive dimensions. In addition, the analysis considers whether academic level, sample size, and measurement instrument type are associated with variation in effect sizes. Through these analyses, the study seeks to clarify how different forms of technological support relate to intercultural learning outcomes. The findings may also offer practical suggestions for intercultural education across different life stages. Distinct from previous syntheses that grouped emerging technologies into broader categories, the present study differentiates AI-, AR-, and VR-supported interventions and examines their effects across cognitive, attitudinal, and behavioral outcomes. This finer analytical approach provides more specific evidence on how different technological forms are associated with different dimensions of intercultural learning. The following research questions guided this study:

RQ1. What is the overall effect of emerging technology-supported intercultural learning on learners’ intercultural outcomes?

RQ2. Do the effects of intercultural learning differ between AR- and VR-based interventions?

RQ3. What is the effect of AI-supported interventions on intercultural learning outcomes?

RQ4. Do outcome dimensions influence the effects of emerging technology-supported intercultural learning?

RQ5. Does academic level influence the effects of emerging technology-supported intercultural learning?

RQ6. Does sample size influence the effects of emerging technology-supported intercultural learning?

RQ7. Does measurement instrument type influence the effects of emerging technology-supported intercultural learning?

## 2. Materials and Methods

### 2.1. Search Strategy

This three-level meta-analysis examined the effects of emerging technology-supported interventions on intercultural learning outcomes. The review process followed the Preferred Reporting Items for Systematic Reviews and Meta-Analyses (PRISMA 2020) framework (Page et al., 2021). PRISMA guided study identification, screening, eligibility assessment, and final inclusion. It also helped report the number of records identified, removed, screened, excluded, and included. We conducted a systematic search in Web of Science, Scopus, and ProQuest, selecting these databases because they cover education, psychology, applied linguistics, communication, and technology-enhanced learning. The searches were updated before manuscript submission, with the final search of all three databases conducted on 31 July 2026. The search included empirical studies published in English.

The search strategy was guided by the PICO framework, which comprises population, intervention, comparison, and outcome (Richardson et al., 1995; Schardt et al., 2007). The population was learners in intercultural, cross-cultural, or language and culture learning contexts. The intervention was emerging technology-supported intercultural learning, including AI, AR, and VR-based interventions. The comparison condition included traditional instruction, learning without these technologies, or alternative instruction. The outcomes were intercultural learning outcomes coded as Cognitive, Attitudinal, or Behavioral. Cognitive outcomes included cultural knowledge, intercultural understanding, and awareness of cultural norms, values, and practices. Attitudinal outcomes included openness, respect, intercultural sensitivity, motivation, and willingness to engage with other cultures. Behavioral outcomes included intercultural communication behavior, adaptive responses, interaction skills, and culturally appropriate problem solving.

The search strategy included two core groups of terms. The first group focused on intercultural learning. It covered intercultural education, intercultural competence, intercultural communication, intercultural awareness, intercultural sensitivity, intercultural skills, cultural competence, CQ, and global competence. The second group focused on emerging technologies. It covered AI, generative AI, ChatGPT, chatbots, conversational agents, intelligent tutoring systems, large language models, AR, VR, XR, MR, immersive technologies, virtual environments, and the metaverse. The search strategies were adapted to the search syntax, field options, and retrieval characteristics of each database. Although the core concepts of intercultural learning and emerging technologies were retained, the scope of search terms and additional restrictions varied across databases, as detailed in Appendix A.

### 2.2. Eligibility Criteria

**Figure 1:**
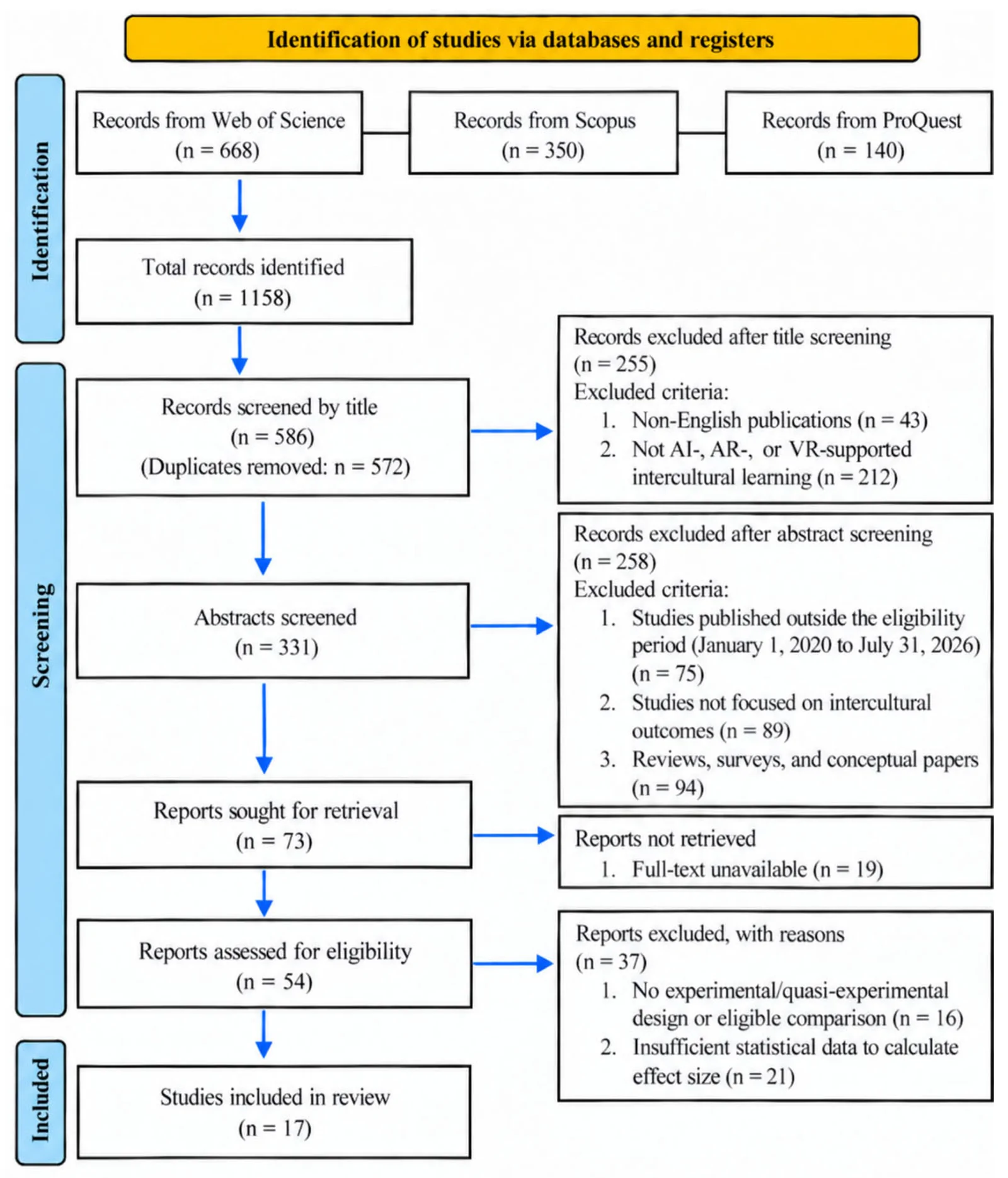
Article Selection Flowchart.

Figure 1 shows that studies were included if they met the following criteria. First, the study examined learners in an educational, language learning, or training context. Second, the intervention used AI, AR, or VR as a core instructional or learning support. Studies that used technology only for administrative delivery or data collection were not included. Third, the study focused on intercultural learning or closely related constructs, such as intercultural competence, CQ, intercultural sensitivity, intercultural communication, cultural knowledge, or intercultural attitudes. Fourth, the study adopted either a randomized experimental design or a quasi-experimental design, allowing the intervention effect to be estimated. Fifth, the study provided sufficient statistical information to calculate an effect size, such as means, standard deviations, and sample sizes, or other statistics that could be converted into a standardized mean difference. Sixth, only studies published in English were eligible. Seventh, studies published from 1 January 2020 to 31 July 2026, were eligible for inclusion. The completed PRISMA 2020 checklist is provided in the Supplementary Materials.

Studies were excluded if they were conceptual papers, theoretical discussions, the literature reviews, qualitative studies without extractable quantitative outcomes, or studies without an independent comparison group or sufficient data to calculate between-group effect sizes. Studies were also excluded if the intervention did not involve AI, AR, or VR, if the outcome was not related to intercultural learning, or if the same dataset was reported in multiple publications without additional eligible outcomes. When duplicate reports appeared to use the same participants and outcomes, the most complete report was retained to avoid double counting. In addition, publication status was not used as an exclusion criterion. Both peer-reviewed studies and preprints were eligible if they met the other inclusion criteria. At the time of the final search, J. Sun and Nie (2026) was the only included study available as a preprint, while the remaining included studies had been published in peer-reviewed outlets. As of October 2026, no peer-reviewed journal version of J. Sun and Nie (2026) could be verified.

### 2.3. Study Selection and Data Extraction

Following duplicate removal, two reviewers independently screened the records by title and subsequently by abstract. Potentially eligible full-text articles were then retrieved and assessed against the prespecified inclusion and exclusion criteria. Data from included studies were independently extracted using the predefined coding framework, including study characteristics, sample sizes, intervention features, outcome measures, and statistical information required for effect-size calculation. Disagreements during study selection or data extraction were resolved through discussion and, when necessary, consultation with a third reviewer.

### 2.4. Coding of Studies and Effect Sizes

As shown in Table 2, studies were coded according to a predefined coding framework before data extraction. The coding framework was informed by previous meta-analytic studies on technology-supported learning and intercultural learning (Shadiev et al., 2026; X. Zhang & Zhou, 2023). Two reviewers independently coded all 17 included studies using the predefined coding framework. Inter-rater agreement was assessed for the categorical variables, including technology type, outcome domain, academic level, sample size category, and measurement instrument type, and was high (Cohen’s κ = 0.96). Any disagreements were resolved through discussion and, when necessary, consultation with a third reviewer. It included five moderator variables: technology type, outcome domain, academic level, sample size, measurement instrument type. Appendix B provides a more detailed study-level overview of the research purposes and key findings of the 17 included studies.

Technology was coded according to the main form of technological support used in each study. AR- and VR-based interventions were distinguished as two types of immersive learning environments. AR-based interventions referred to studies in which digital cultural information, visual prompts, or interactive objects were integrated into the physical learning environment. VR-based interventions referred to studies using immersive or simulated virtual environments to create intercultural scenarios, communication contexts, or culturally situated learning experiences (Shadiev et al., 2026). Throughout this review, emerging technologies refers collectively to AI, AR, and VR. XR is used only as an umbrella term for AR and VR, not as an additional analytical category.

AI-supported interventions were coded separately. These included studies using AI systems, chatbots, generative AI tools, intelligent agents, or adaptive communication systems to support interaction, feedback, dialogue, or personalized learning. When more than one technological component was used, coding was based on the component that played the primary instructional role in relation to the reported intercultural outcomes. The study by W. Wu and Sun (2025) was classified as AI-supported based on the original study’s description of Mursion as integrating AI technology and personalized feedback with human-operated avatars.

As shown in Table 3, outcome domains were coded as Cognitive, Attitudinal, or Behavioral by adapting the cognitive, affective, and behavioral classification used by Fukkink et al. (2024). The affective domain was operationalized more narrowly as Attitudinal to distinguish evaluative dispositions toward cultural differences from general emotional or motivational states. Classification was based on construct definitions, item content, and scoring criteria rather than outcome labels. Cognitive outcomes assessed cultural knowledge, recognition, or understanding. Cultural awareness was coded as Cognitive when it reflected recognition or understanding and as Attitudinal when it reflected evaluation, openness, or acceptance. Attitudinal outcomes included intercultural sensitivity, openness, respect, empathy, and motivation explicitly directed toward intercultural engagement. General learning motivation and engagement were excluded, and anxiety was not coded as Attitudinal because it represents an affective state rather than an evaluative disposition. Behavioral outcomes included intercultural communication, adaptation, culturally appropriate performance, and behavioral intentions; willingness to communicate was therefore coded as Behavioral. Speaking and pragmatic performance were classified as Behavioral only when their scoring criteria explicitly assessed intercultural interaction or cultural appropriateness rather than general language proficiency. Engagement was included only when it represented observable participation in intercultural interaction. Measures combining multiple domains without separable scores were excluded from domain-specific analyses.

Academic level was coded as K–12 learners or adult learners. K–12 learners included primary and secondary school students. Adult learners included university students, preservice teachers, in-service teachers, and adult participants in learning contexts. This distinction was used because younger learners and adult learners may differ in cognitive maturity, prior intercultural experience, autonomy, and familiarity with digital tools (Shadiev et al., 2026).

Study sample size was examined as an exploratory moderator. Following Talan et al. (2020), studies with fewer than 50 participants were classified as small, those with 50–99 participants as medium, and those with 100 or more participants as large. Using an existing classification scheme helped avoid selecting cutoffs based on the present data and facilitated comparisons across studies of different sizes. These thresholds were treated as operational categories rather than as clear points at which intervention effects were expected to change. Given that only 17 independent studies were included and several study characteristics overlapped, sample size was not further analyzed as a continuous moderator. The sample-size findings should therefore be regarded as exploratory.

Intervention duration was recorded descriptively. Of the 17 included studies, three interventions lasted ≤4 weeks, five lasted 5–8 weeks, and six lasted >8 weeks; duration was unclear for three studies. Intervention duration was not examined as a moderator because of the limited number of studies in each category and incomplete reporting.

Following the distinction highlighted by X. Zhang and Zhou (2023), measurement instrument type was coded at the effect-size level as Established, Adapted, or Unclear. Established measures were previously published instruments with prior evidence of reliability or validity and were used without substantive modification in the included study. Adapted measures included instruments developed specifically for the study or existing instruments that were substantially modified for the study context. Translation or the use of a previously validated localized version alone was not considered a substantive adaptation. Measures with insufficient information for classification were coded as Unclear and excluded from the moderator analysis. Comparator conditions were descriptively classified as technology-based or conventional or nontechnology based on the learning activities provided to the comparison groups. Three studies used technology-based comparators, whereas the remaining 14 used conventional or nontechnology comparators.

### 2.5. Prespecified Research and Analytical Decisions

To enhance methodological transparency, we specify the research and analytical decisions that were established prior to data extraction and formal statistical analysis. The research questions concerning the overall effect of emerging technology-supported intercultural learning, technology type, outcome domain, academic level, and sample size were determined in advance, together with the corresponding coding framework. The analysis of measurement instrument type (RQ7) was introduced during manuscript revision to further examine methodological differences across the included studies. Technology type was classified as AI, AR, or VR, outcome measures were categorized as cognitive, attitudinal, or behavioral, and academic level was coded according to predefined learner groups. Sample size was also classified a priori as small (<50), medium (50–99), or large (≥100), following Talan et al. (2020). These thresholds were adopted from previous research rather than derived from the observed distribution of the included studies or the results of the moderator analyses. The planned analyses included the overall three-level random-effects model, the comparison between AR- and VR-based interventions, the separate estimation of AI-supported interventions, and moderator analyses for outcome domain, academic level and sample size. Sensitivity analyses were conducted to assess the robustness of the pooled estimates under alternative within-study correlation assumptions, after excluding extreme effect sizes (g > 4.0), and after excluding studies judged to be at serious risk of bias. An additional post hoc sensitivity analysis applied a threshold of g > 2.5. The AR leave-one-out analysis and the sensitivity analysis excluding J. Sun and Nie (2026), the only preprint, were conducted as additional post hoc sensitivity analyses and were not prespecified. The eligibility period covering 2020–2026 was established from the outset of the review. The final database searches were conducted on 31 July 2026, without extending the originally defined eligibility period.

### 2.6. Data Analyses

For each outcome, the effect size was calculated as Hedges’ g. This index shows the standardized difference between the technology-supported intervention group and the comparison group. Post-intervention means, standard deviations, and sample sizes were used when available. All effect sizes were based on between-group comparisons, with no single-group pre–post effects included. For quasi-experimental studies, baseline comparability was considered using available pretest information and risk-of-bias assessments, while effect sizes were primarily calculated from between-group posttest means and standard deviations rather than covariate-adjusted estimates. If a study reported other convertible statistics, they were transformed into standardized mean differences when possible. All effect sizes were coded in the same direction, with positive values showing better intercultural learning outcomes in the technology-supported group.

A three-level random-effects model was used because several studies reported multiple effect sizes. The model was estimated using restricted maximum likelihood (REML) in the R (version 4.6.1; R Foundation for Statistical Computing, Vienna, Austria) with the metafor package (version 5.0-1; Viechtbauer, 2010), with random effects specified at the study and effect-size levels to account for hierarchical clustering and heterogeneity. A 95% prediction interval was calculated for the overall pooled effect to represent the range of effects that might be expected in a new comparable study, taking the estimated heterogeneity into account. The primary model used a diagonal sampling variance matrix containing the estimated variance of each effect size. This working specification did not model correlated sampling errors among outcomes from the same participants, because the required outcome correlations were not consistently reported. Study-level and effect-size-level random effects account for hierarchical heterogeneity but do not, by themselves, remove correlated sampling errors. To further examine the influence of within-study dependence, variance-covariance matrices were subsequently constructed under different assumed correlations and evaluated in sensitivity analyses. In Knutzen et al. (2025), the different sample sizes reflected outcome-specific response availability within the same participant groups rather than independent samples; these outcomes were therefore treated as multiple effect sizes nested within the same study. Thus, sample independence was not modeled as an additional random-effects level. Experimental and control groups denote participant arms within primary studies, whereas subgroups denote categories used in moderator analyses. Given the limited number of independent studies (n = 17), all moderator analyses were considered exploratory. Their results were interpreted cautiously because of the limited statistical power to detect differences between subgroups. Cluster-robust variance estimation with small-sample corrections was considered as an alternative inferential approach but was not implemented in the present analysis. Given the limited number of independent studies and the uneven distribution of studies across moderator categories, particularly those represented by only two studies, such corrections would not fully resolve the limitations associated with sparse subgroup evidence. We therefore retained the three-level random-effects model as the primary analytical framework and interpreted moderator tests as exploratory rather than confirmatory.

### 2.7. Risk of Bias Assessment

Risk of bias was assessed according to study design. Randomized studies were evaluated using the revised Cochrane Risk of Bias tool (RoB 2; Sterne et al., 2019), whereas non-randomized studies were evaluated using the Risk of Bias in Non-randomized Studies of Interventions tool (ROBINS-I; Sterne et al., 2016). Two reviewers independently assessed each study at the domain and overall levels. Disagreements were resolved through discussion and, when necessary, consultation with a third reviewer. Publication status was recorded during study selection but was not used to determine study eligibility. The preprint was therefore included and assessed in the same way as peer-reviewed studies. Its risk of bias was evaluated using the appropriate tool for its study design and was based on the methodological information reported in the study, rather than on its publication status.

### 2.8. Sensitivity Analysis and Assessment of Publication Bias

Sensitivity analyses were conducted to examine whether the main results were stable. The overall effect was recalculated after checking for potentially influential effect sizes and studies that contributed multiple outcomes. The results were then compared with the main analysis to see whether the direction and significance of the effect changed. To examine the sensitivity of the results to potential correlated sampling errors that were not explicitly modeled in the primary analysis, sampling variance-covariance matrices (V) were constructed using assumed common within-study correlations (ρ) of 0.30, 0.40, 0.50, 0.60, and 0.70, and the three-level model was refitted at each value. These analyses were intended to assess whether the main conclusions depended on plausible assumptions about within-study sampling dependence. An additional analysis excluded all eight effect sizes from J. Sun and Nie (2026) and refitted the same overall model to the remaining 16 independent studies and 69 effect sizes. The AI subgroup model was also refitted after excluding J. Sun and Nie (2026), leaving eight independent studies and 35 effect sizes. Publication bias and small study effects were assessed using funnel plots, Egger’s regression test, and Begg’s rank correlation test. For these diagnostics, multiple effect sizes were summarized at the study level so that studies, rather than individual dependent effect sizes, formed the units used in the bias assessment. Funnel plots were inspected to see whether effect sizes were evenly distributed around the pooled effect. Egger’s and Begg’s tests were used to examine whether smaller or less precise studies showed different effects. Non-significant results were interpreted as showing no strong statistical evidence of publication bias or small-study effects, rather than as proof that bias was absent. These covariance-based sensitivity analyses were restricted to the overall pooled effect. They were not extended to moderator analyses because several moderator categories contained very few independent studies, limiting the interpretability of subgroup-specific sensitivity estimates. Consequently, the stability of moderator conclusions under alternative within-study correlation assumptions was not established.

## 3. Results

### 3.1. Overall Effect Size (RQ1)

**Figure 2:**
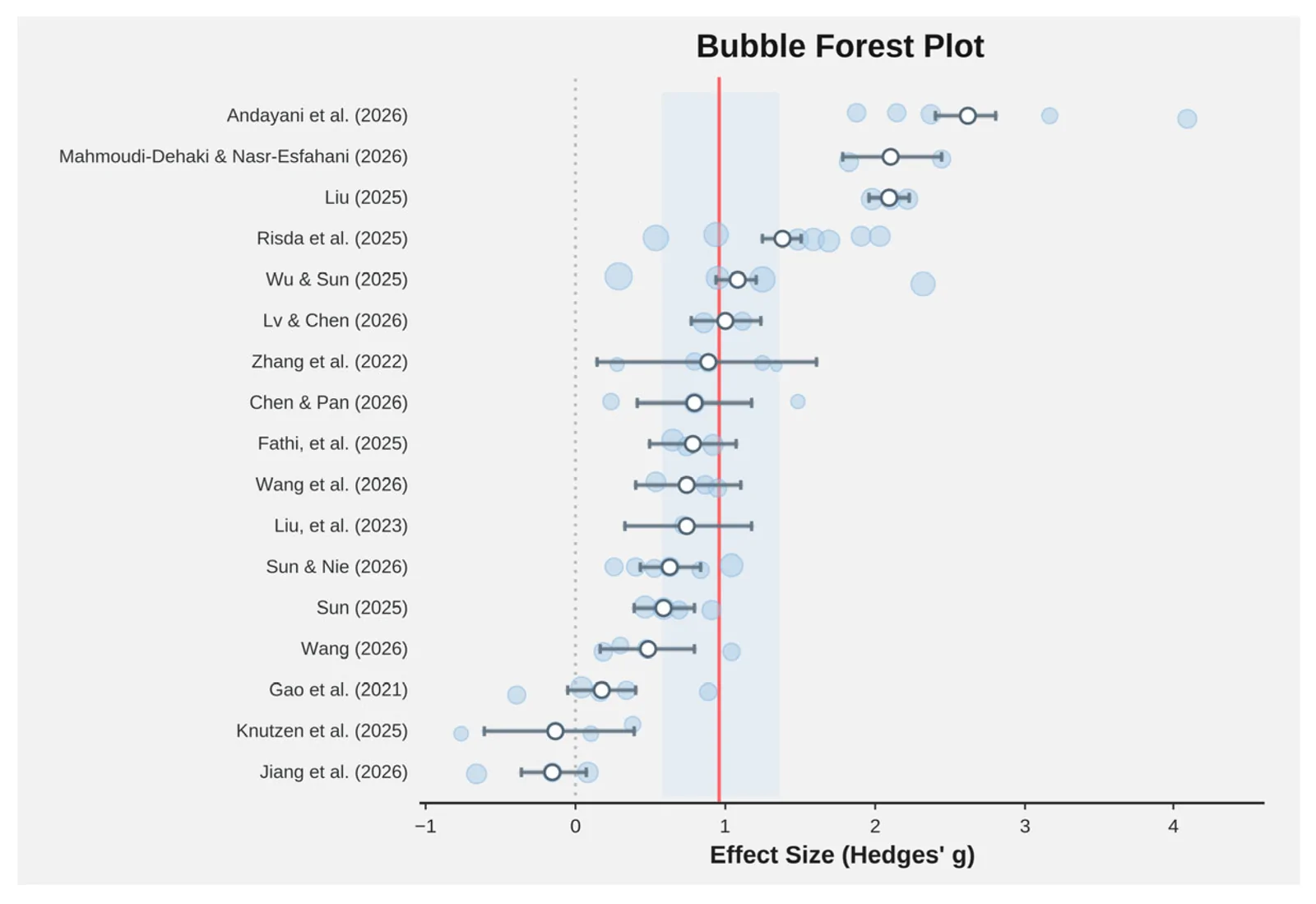
Forest Plot. The light-blue circles represent individual effect sizes; the open circles and horizontal error bars show study-level estimates and their 95% confidence intervals. The solid red line shows the overall pooled effect (g = 0.965), the pale vertical band shows its 95% confidence interval, and the grey dotted line marks g = 0. The study labels correspond to Gao et al. (2021), B. Zhang et al. (2022), S. Liu et al. (2023), Fathi et al. (2025), L. Sun (2025), J. Liu (2025), W. Wu and Sun (2025), Risda et al. (2025), Knutzen et al. (2025), Chen and Pan (2026), J. Sun and Nie (2026), Z. Wang (2026), Y. Wang et al. (2026), Lv and Chen (2026), Mahmoudi-Dehaki and Nasr-Esfahani (2026), Andayani et al. (2026), and Jiang et al. (2026).

Figure 2 shows the overall effect of technology-supported intercultural interventions. The three-level random-effects model included 77 effect sizes nested within 17 independent studies. The pooled effect was statistically significant, g = 0.965, 95% CI [0.580, 1.350], *t* (76) = 4.997, *p* < .001. The 95% prediction interval was approximately [−0.781, 2.711], indicating substantial variability in the effects that might be observed in a new comparable study. Although the average effect was positive, the prediction interval crossed zero, suggesting that the effect in an individual future study may range from negative or negligible to strongly positive. As shown in Figure 2, most study-level effects were positive, although effect sizes varied across studies. The pooled estimate applies to the included AI-, AR-, and VR-supported interventions.

**Figure 3:**
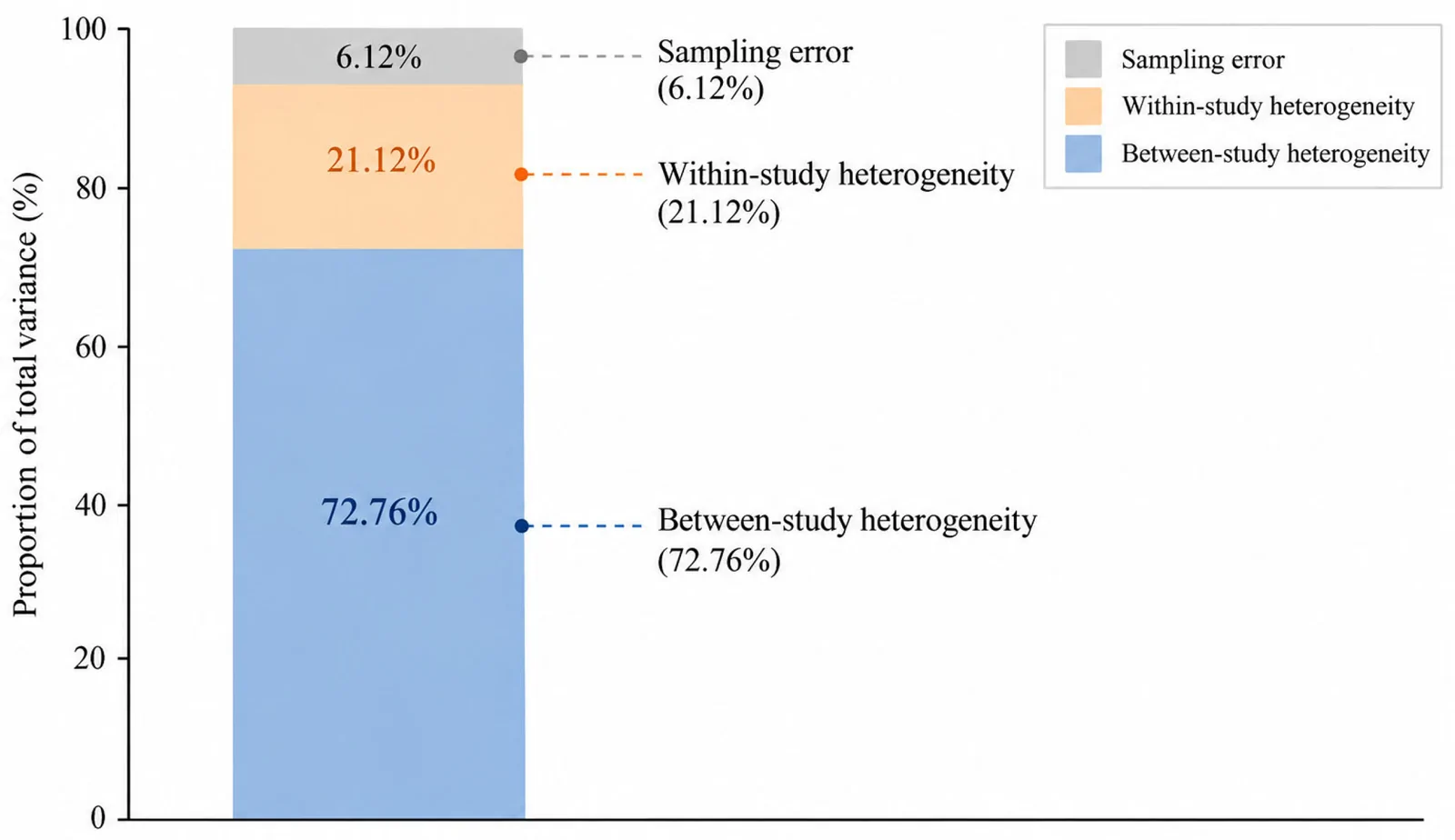
Variance Decomposition in the Three-Level Meta-Analytic Model.

Figure 3 presents the variance decomposition of the three-level random-effects model. The heterogeneity test was statistically significant, *Q* (76) = 1118.147, *p* < .001, indicating substantial variability among the included effect sizes. The estimated variance components showed that heterogeneity existed at both the study level (*σ*^2^ = 0.566) and the effect-size level (*σ*^2^ = 0.164). The corresponding I^2^-like variance decomposition indicated that 72.76% of the total variance was attributable to between-study heterogeneity and 21.12% to within-study heterogeneity, yielding a total I^2^ of 93.88%; the remaining 6.12% reflected sampling error. This suggested that variation across studies exceeded variation among effect sizes within the same study. In other words, differences between studies were the main source of heterogeneity. This substantial heterogeneity suggests that the pooled effect may not be generalizable across different educational contexts and technological interventions, and the findings should therefore be interpreted with caution. Therefore, subsequent moderator analyses examined whether outcome domain, academic level, sample size, and measurement instrument type explained variation in effect sizes.

### 3.2. AR- and VR-Based Interventions (RQ2)

Table 4 presents the comparison between AR- and VR-based interventions. The analysis included 34 effect sizes from eight independent studies: two AR studies contributed 10 effect sizes, and six VR studies contributed 24 effect sizes. The moderator test showed a significant difference between AR- and VR-based interventions, *F* (1, 32) = 6.315, *p* = .017. AR was used as the reference group and showed a positive estimated effect, g = 1.856, 95% CI [0.820, 2.893], *p* < .001. The two AR studies showed notably different model-based estimates: S. Liu et al. (2023), g = 0.745, 95% CI [−0.254, 1.744], and Andayani et al. (2026), g = 2.767, 95% CI [1.957, 3.577]. Compared with AR, VR-based interventions showed a significantly smaller estimated effect, *β* = −1.481, 95% CI [−2.681, −0.280], *p* = .017. Residual heterogeneity remained significant, *QE* (32) = 186.360, *p* < .001, indicating that other study characteristics may still explain variation in effect sizes. However, the AR subgroup included only two independent studies, and the pooled AR estimate was strongly influenced by Andayani et al. (2026). The AR–VR difference was no longer statistically significant after excluding this study, indicating that the current evidence does not support a definitive conclusion that AR is more effective than VR.

### 3.3. AI-Supported Interventions (RQ3)

Table 5 presents the overall effect of AI-supported intercultural learning. The analysis included 43 effect sizes from nine independent studies. AI-supported interventions showed a significant positive effect, g = 1.150, 95% CI [0.744, 1.555], *p* < .001. Significant heterogeneity was also observed, *Q* (42) = 537.709, *p* < .001. This result indicates that AI-supported intercultural learning was generally associated with positive learning outcomes. However, the significant heterogeneity suggests that the magnitude of the effect varied across studies and may depend on differences in intervention design, learning context, or other study characteristics. The 95% prediction interval was approximately [−0.290, 2.589]. The estimated variance components were 0.312 at the study level and 0.157 at the effect-size level. The corresponding I^2^-like variance decomposition indicated that 61.95% of the total variance was attributable to between-study heterogeneity and 31.08% to within-study heterogeneity, while the remaining 6.97% reflected sampling error.

### 3.4. Outcome Domain (RQ4)

Table 6 presents the moderating effect of outcome domain. The moderator test was not statistically significant, *F* (2, 74) = 1.781, *p* = .176, indicating that the effects did not differ reliably across cognitive, attitudinal, and behavioral outcomes. The cognitive reference group showed a significant positive effect, g = 0.892, 95% CI [0.469, 1.315], *p* < .001. Neither attitudinal outcomes (*β* = −0.028, *p* = .858) nor behavioral outcomes (*β* = 0.239, *p* = .110) differed significantly from cognitive outcomes. Cognitive outcomes were represented by 14 independent studies and 27 effect sizes, attitudinal outcomes by 11 studies and 27 effect sizes, and behavioral outcomes by 15 studies and 23 effect sizes.

### 3.5. Academic Level (RQ5)

Table 7 presents the moderating effect of academic level. The moderator test was statistically significant, *F* (1, 75) = 17.27, *p* < .001. Adult learners were used as the reference group and showed a positive estimated effect, g = 0.758, 95% CI [0.469, 1.047]. The estimated effect for K–12 learners was larger than that for adult learners, *β* = 1.679, 95% CI [0.874, 2.484]. Residual heterogeneity remained substantial, *QE* (75) = 689.309, *p* < .001, suggesting that additional study characteristics may account for variation in the observed effects. The adult category comprised 15 independent studies and 65 effect sizes, whereas the K–12 category comprised only two independent studies and 12 effect sizes. Given the limited K–12 evidence, this moderator finding should be regarded as exploratory.

### 3.6. Sample Size (RQ6)

Table 8 presents the moderating effect of sample size. The moderator test was statistically significant, *F* (2, 74) = 3.873, *p* = .026. This result suggests that the effect of emerging technology-supported intercultural learning differed by sample size. Studies with large samples were used as the reference group. This group showed a significant positive effect, g = 1.575, 95% CI [1.029, 2.121], *p* < .001. Compared with large-sample studies, medium-sample studies showed a significantly smaller effect, *β* = −0.958, 95% CI [−1.730, −0.186], *p* = .016. Small-sample studies also showed a significantly smaller effect, *β* = −0.960, 95% CI [−1.802, −0.117], *p* = .026. These results indicate that larger-sample studies reported stronger intervention effects than medium- and small-sample studies. Residual heterogeneity remained significant, *QE* (74) = 861.335, *p* < .001, suggesting that other study-level factors may still explain differences in effect sizes. The large-sample category comprised six independent studies and 30 effect sizes, the medium-sample category six studies and 32 effect sizes, and the small-sample category five studies and 15 effect sizes. A cross-tabulation of sample size by technology type showed that the small-sample group comprised one AI, one AR, and three VR studies; the medium-sample group comprised four AI, no AR, and two VR studies; and the large-sample group comprised four AI, one AR, and one VR study. These distributions indicate that sample size was not evenly distributed across technology types. Given the limited number of independent studies (n = 17) and the uneven distribution across moderator categories, additional multivariable and within-AI subgroup analyses were not conducted due to concerns about estimate stability. Although sample size was categorized using previously established thresholds, its sensitivity to a continuous specification, such as log (total n), was not examined. Therefore, the observed sample-size moderation may be confounded by technology type and academic level and should be interpreted as exploratory. Sensitivity analyses excluding W. Wu and Sun (2025) yielded a nearly identical pooled AI effect (g = 1.147, 95% CI [0.679, 1.616], *p* < .001) compared with the original estimate (g = 1.150). The sample-size moderator also remained significant, *F* (2, 70) = 3.842, *p* = .026.

### 3.7. Measurement Instrument Type (RQ7)

Table 9 presents the moderating effect of measurement instrument type. The moderator test was not statistically significant, *F* (1, 72) = 1.547, *p* = .218. This result suggests that the effect of emerging technology-supported intercultural learning did not differ significantly by measurement instrument type. Studies using established measures were used as the reference group. This group showed a significant positive effect, g = 0.858, 95% CI [0.399, 1.317], *p* < .001. Compared with established measures, studies using adapted measures showed a numerically larger effect, *β* = 0.296, 95% CI [−0.178, 0.770], *p* = .218, but the difference was not statistically significant. Residual heterogeneity remained significant, *QE* (72) = 975.226, *p* < .001, suggesting that other factors may still explain differences in effect sizes. The established-measure category comprised 10 independent studies and 36 effect sizes, whereas the adapted-measure category comprised nine studies and 38 effect sizes. Three effect sizes with unclear measurement instrument type status were excluded from this moderator analysis.

Because technology type, academic level, and sample size were examined in separate moderator analyses, the distribution of these study characteristics was further inspected to assess potential overlap among them. Among the six large-sample studies, four examined AI-supported interventions, one examined AR, and one examined VR. In addition, both K–12 studies were classified as large-sample studies, with one using AI and the other using AR. These distributions indicate that academic level and sample size were not evenly distributed across technology types. Therefore, the significant moderator effects observed for academic level and sample size may partly reflect differences in the composition of the included studies rather than independent effects of these characteristics. This overlap should be considered when interpreting the corresponding moderator findings.

### 3.8. Publication Bias

**Figure 4:**
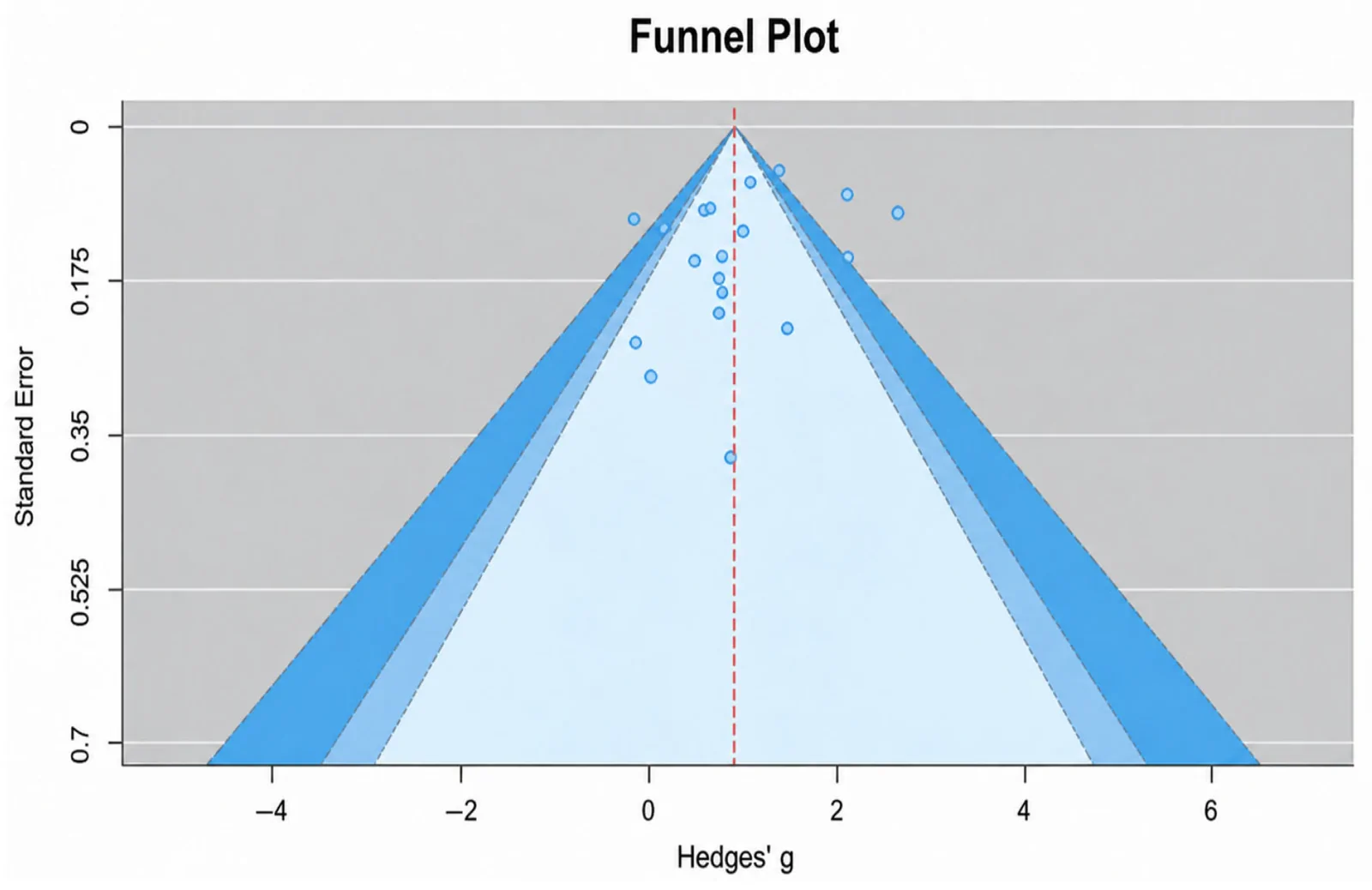
Funnel Plot of Study-Level Hedges’ g Estimates. Blue circles represent study-level estimates, blue shaded triangular bands represent reference contours, and the red dashed vertical line marks the overall pooled effect (g = 0.965).

Figure 4 presents the funnel plot for publication bias assessment based on 17 study-level effect estimates. Most study-level effects were located within the expected funnel region and were generally distributed around the pooled effect. Visual inspection did not indicate marked funnel-plot asymmetry. Egger’s regression test (n = 17) was not statistically significant, *z* = −1.107, *p* = .268. Begg’s rank correlation test (n = 17) was also not significant, Kendall’s τ = −0.044, *p* = .839. These results suggest no strong statistical evidence of publication bias or small-study effects. However, given the limited number of independent studies (n = 17), Egger’s and Begg’s tests may have had insufficient statistical power to detect publication bias. Therefore, non-significant results should not be interpreted as evidence that publication bias was absent.

### 3.9. Risk of Bias

**Figure 5:**
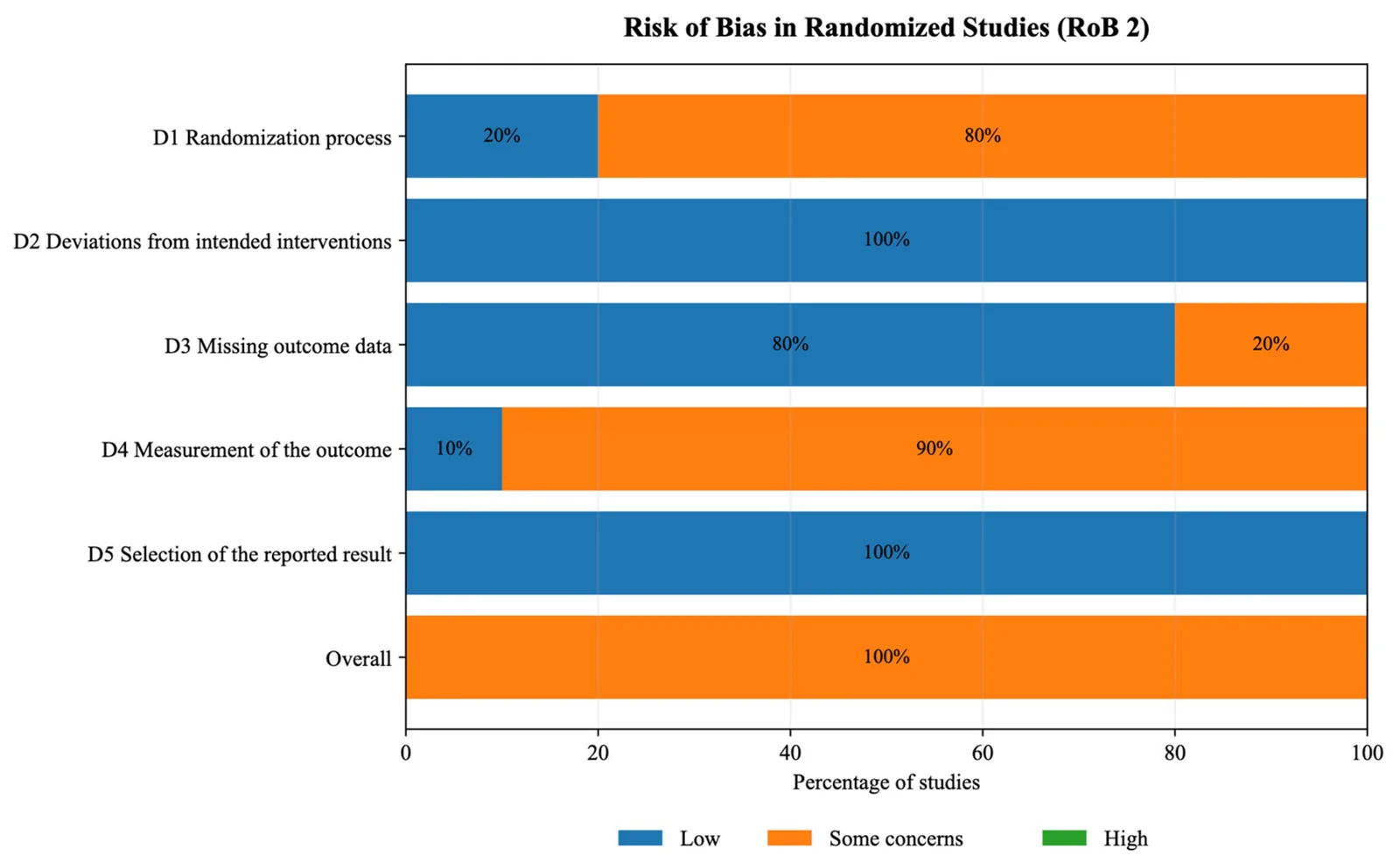
Summary risk-of-bias assessment of randomized studies using RoB 2. Green (high risk) is retained in the legend for completeness, although no included randomized studies were rated as high risk overall.

**Figure 6:**
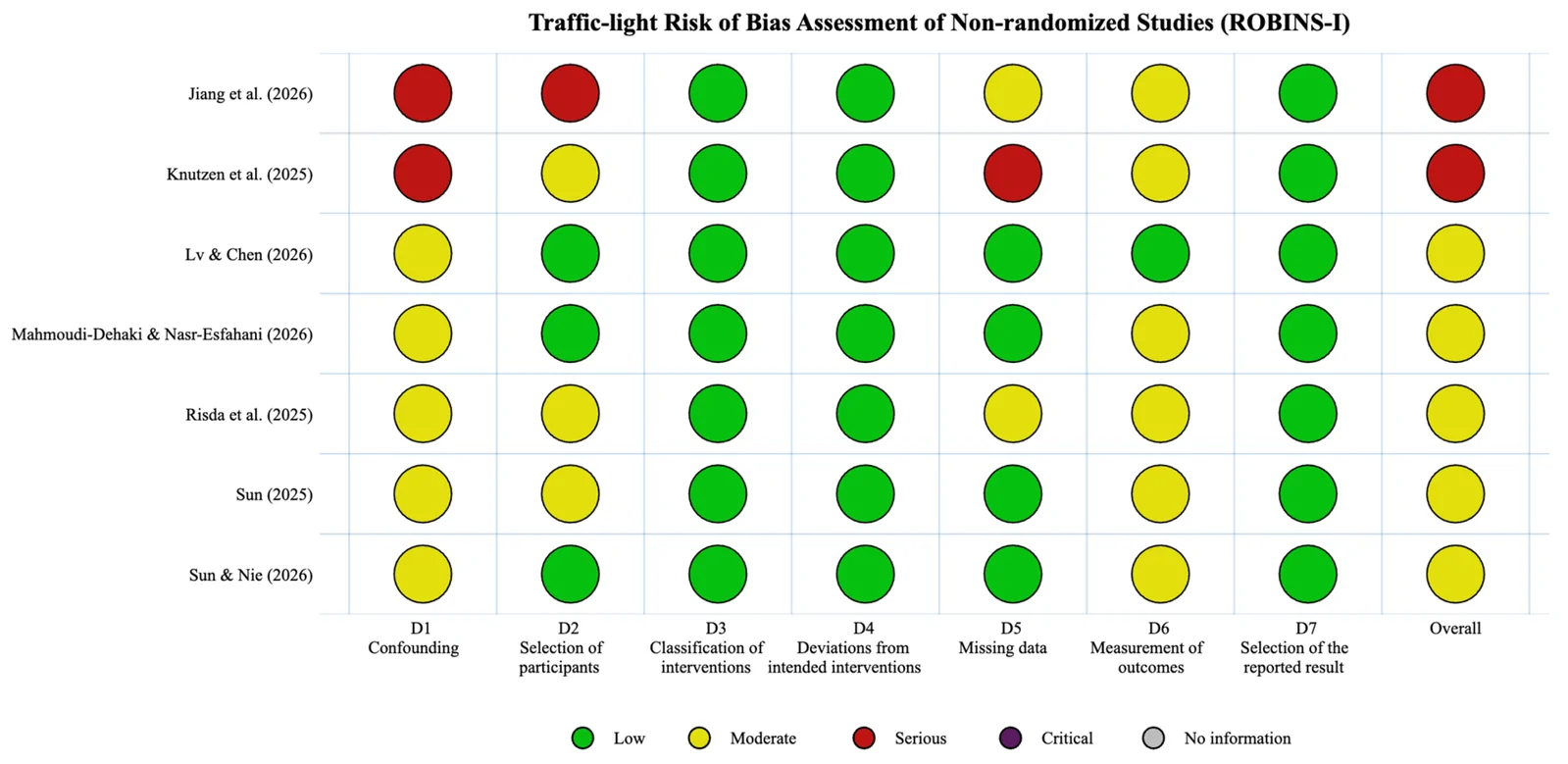
Traffic-light risk-of-bias assessment of non-randomized studies using ROBINS-I. Purple (critical risk) and gray (no information) are retained in the legend for completeness, although no included studies were assigned to these categories. Study labels correspond to Knutzen et al. (2025), Risda et al. (2025), L. Sun (2025), Jiang et al. (2026), Lv and Chen (2026), Mahmoudi-Dehaki and Nasr-Esfahani (2026), and J. Sun and Nie (2026).

As shown in Figure 5, all randomized studies were judged as having some concerns overall under RoB 2, with no study rated as high risk. The main concerns were related to the randomization process and outcome measurement. As shown in Figure 6, five of the seven non-randomized studies were judged to have a moderate overall risk of bias, while two studies, Jiang et al. (2026) and Knutzen et al. (2025), were rated as serious, mainly because of concerns regarding confounding, participant selection, and outcome measurement.

### 3.10. Sensitivity Analysis

**Figure 7:**
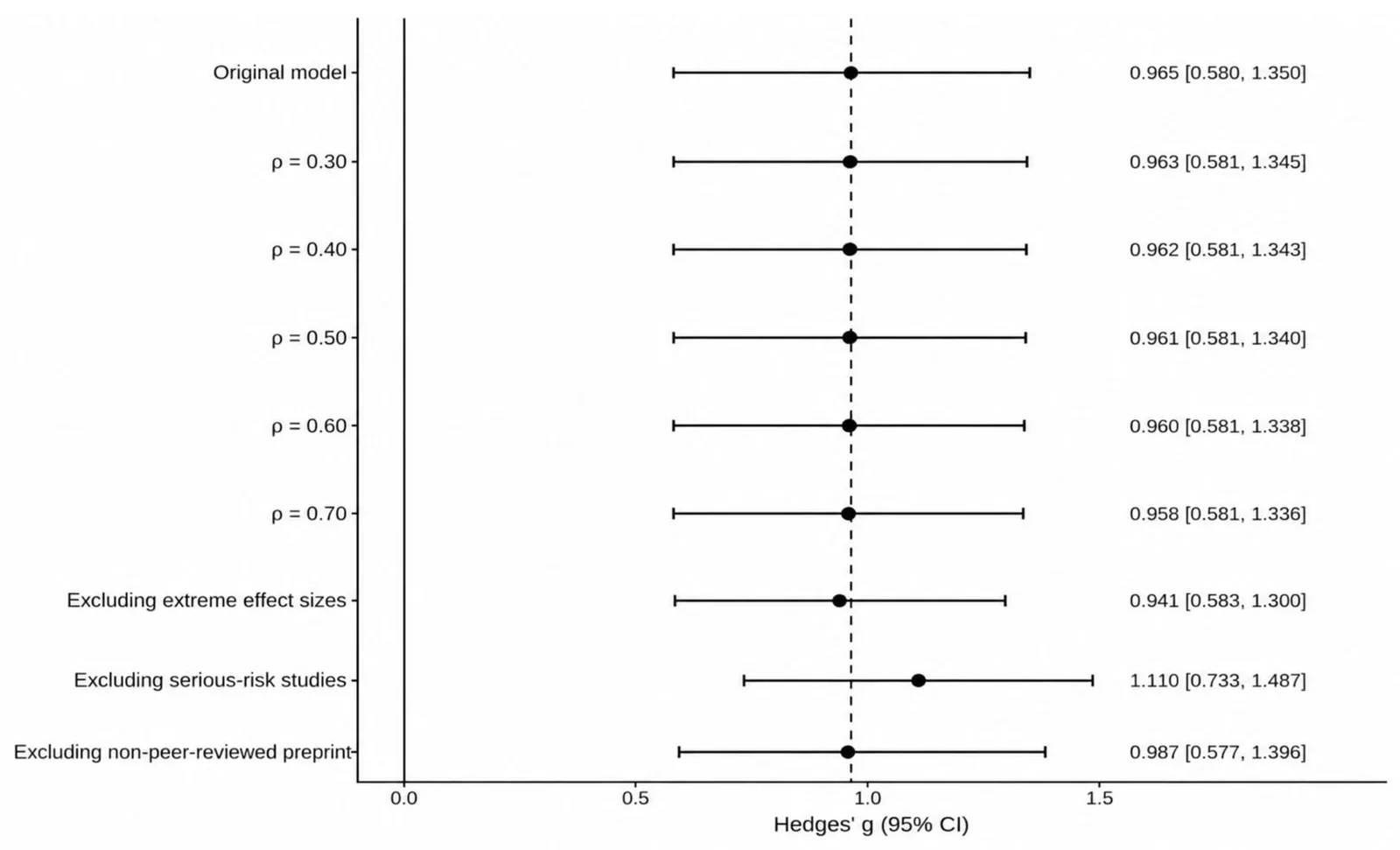
Sensitivity analyses of the overall pooled effect. The vertical dashed line marks the original pooled effect (g = 0.965); horizontal error bars indicate 95% confidence intervals.

Figure 7 presents the sensitivity analysis results. The original three-level model yielded a significant pooled effect, g = 0.965, 95% CI [0.580, 1.350], *p* < .001. Varying the assumed within-study correlation from ρ = 0.30 to ρ = 0.70 produced highly similar estimates. Specifically, the pooled effects were g = 0.963, 95% CI [0.581, 1.345] at ρ = 0.30; g = 0.962, 95% CI [0.581, 1.343] at ρ = 0.40; g = 0.961, 95% CI [0.581, 1.340] at ρ = 0.50; g = 0.960, 95% CI [0.581, 1.338] at ρ = 0.60; and g = 0.958, 95% CI [0.581, 1.336] at ρ = 0.70. After excluding two extreme effect sizes (g > 4.0) from Andayani et al. (2026), corresponding to symbol identification (g = 4.091) and behavioral time-on-task (g = 4.098), the pooled effect remained similar, g = 0.941, 95% CI [0.583, 1.300]. An additional post hoc sensitivity analysis using a threshold of g > 2.5 also excluded tradition explanation (g = 3.165), yielding a comparable pooled effect, g = 0.932, 95% CI [0.583, 1.282]. The extreme effect sizes were verified using the reported group means, standard deviations, and sample sizes. The nearly identical estimates across the assumed correlations (ρ = 0.30–0.70) suggest that the main findings were not sensitive to the diagonal sampling variance specification used in the primary model. After excluding Jiang et al. (2026) and Knutzen et al. (2025), the pooled effect remained significant and increased slightly, g = 1.110, 95% CI [0.733, 1.487], because these studies contributed relatively small or negative effects. A further sensitivity analysis excluded J. Sun and Nie (2026), the only non-peer-reviewed preprint included in the review. The pooled effect remained significant and was highly similar to the primary estimate, g = 0.987, 95% CI [0.577, 1.396], *p* < .001. When J. Sun and Nie (2026) was excluded from the AI subgroup, the pooled effect remained significant (g = 1.217, 95% CI [0.777, 1.657], *p* < .001), compared with the original AI estimate (g = 1.150, 95% CI [0.744, 1.555]). This analysis included eight independent studies and 35 effect sizes, supporting the stability of the AI subgroup findings. All confidence intervals remained above zero. For the AR–VR comparison, an additional leave-one-out analysis was conducted because the AR subgroup included only two independent studies. The AR–VR difference remained significant after excluding S. Liu et al. (2023), *β* = −2.409, 95% CI [−3.306, −1.512], *p* < .001, whereas the difference was not statistically significant after excluding Andayani et al. (2026), *β* = −0.387, 95% CI [−1.461, 0.687], *p* = .464. Given the limited AR evidence base, these results further support interpreting the AR–VR comparison as exploratory. Overall, the sensitivity analyses supported the stability of the pooled effect across alternative within-study correlation assumptions and study exclusions. However, this stability should not be generalized to the moderator findings, as the covariance-based sensitivity analyses were restricted to the overall effect. In particular, the AR–VR comparison was sensitive to the exclusion of one AR study. Therefore, the moderator findings should be interpreted as exploratory, given the limited number of independent studies in several categories.

## 4. Discussion

### 4.1. Effects of Emerging Technologies on Intercultural Learning

The present three-level meta-analysis found a strong positive effect of emerging technology-supported intercultural learning. The included studies suggest that emerging technologies may support intercultural learning by providing different opportunities for learners to engage with cultural content. For example, L. Sun (2025) used AI in a 12-week Collaborative Online International Learning (COIL) program between Chinese English majors and Malaysian peers. AI supported translation, communication, feedback and online coordination. These functions reduced the pressure of language problems during cross-national collaboration. Learners could then focus on comparing ideas. They could also complete projects and negotiate cultural meanings. This helps explain the gains in attitudes, intercultural communication skills and intercultural consciousness. A similar pattern appeared in Fathi et al. (2025), where VR scenarios allowed learners to apply cultural understanding and communication strategies in situated tasks. B. Zhang et al. (2022) used VR interactive games with Chinese and Irish students. The VR environment provided avatars, virtual rooms and cultural artifacts. These features created a shared space for students to complete tasks together. The game structure gave learners a concrete reason to communicate. It also encouraged them to negotiate meaning and exchange cultural knowledge. This may explain why the virtual environment supported cultural learning more effectively than text-based assignments. Taken together, the positive effect may be partly explained by how emerging technology changed the learning process. They helped learners communicate across languages, apply cultural understanding to task situations and use cultural knowledge during peer interaction rather than simply presenting cultural knowledge.

The magnitude of the pooled effect should be distinguished from the strength of the underlying evidence. The overall effect was large and remained significant after the two studies at serious risk of bias were excluded. However, the remaining studies were not all at low risk of bias. All randomized studies were rated as having some concerns under RoB 2. The main issues involved the randomization process and outcome measurement. Most non-randomized studies were rated as having moderate risk under ROBINS-I, mainly because of confounding, participant selection, and outcome measurement. The sensitivity analysis shows that the overall finding was not driven only by the two studies at serious risk. The increase in the pooled effect after excluding Jiang et al. (2026) and Knutzen et al. (2025) suggests that these studies attenuated rather than inflated the primary estimate, further supporting the robustness of the overall findings. However, it does not remove the methodological limitations of the remaining studies. Therefore, the evidence supports a positive effect of emerging technology-supported intercultural learning, but the exact size of this effect should be interpreted with caution.

### 4.2. Effects of AR and VR on Intercultural Learning

Although AR showed a larger estimated effect than VR, this difference was not robust to the exclusion of Andayani et al. (2026). Given that the AR subgroup included only two independent studies, this finding should be interpreted as exploratory. AR and VR showed significantly different effects on emerging technology-supported intercultural learning in the current dataset. AR produced the larger estimated mean effect, while VR showed a significantly smaller effect. However, this finding does not establish the general superiority of AR. This is because the AR estimate was based on only two independent studies. The clearest common feature of the two AR studies was their use of physical objects or locations to support the interpretation of cultural meaning. In S. Liu et al. (2023), learners searched their surroundings for cultural artifacts and landmarks. They then connected these visible cues with cultural information presented in the AR application. In Andayani et al. (2026), learners scanned marker cards to activate three-dimensional representations of cultural objects. The largest gain occurred in cultural symbol identification. Cognitive engagement also explained a substantial proportion of the intervention effect. In both studies, the cultural cue was closely connected to what learners located or manipulated, what they were expected to understand, and what was later measured. This connection is important for intercultural learning because cultural meanings are often embedded in artifacts, symbols, gestures, and everyday settings. The large AR estimate may therefore reflect close alignment between visible cultural cues, learner actions, and knowledge-oriented outcome measures. It may not represent a general advantage of AR itself.

The VR studies showed a different pattern. Many interventions created rich and immersive environments, but immersion did not always lead to stronger intercultural learning. Gao et al. (2021), for example, placed learners in a Christmas-themed VR scenario. Learners generally responded positively to the experience. However, VR did not produce significantly better cultural knowledge, behavior, or attitudes than touchscreen learning. B. Zhang et al. (2022) reported a similar pattern. Their contextual games increased English-related discussion and expressions of excitement. However, they did not significantly increase discussion of Irish cultural knowledge or university information. These findings suggest that immersive environments may increase engagement without necessarily directing learners toward the intended cultural content. A similar issue appeared in more open forms of VR. Knutzen et al. (2025) allowed students to create cultural rooms in social VR. This gave learners more freedom to construct and present cultural content. However, the study found no significant advantage over videoconferencing in CQ or motivation. Participants also experienced technical difficulties and problems with platform familiarity and onboarding. Greater immersion and interaction may therefore not be sufficient on their own. Learners still need clear cultural tasks, appropriate guidance, and enough time to become familiar with the platform.

Y. Wang et al. (2026) further showed that some of these limitations can be reduced through sustained and structured implementation. Their six-week program combined preparation, guided VR activities, and post-task reflection. It produced lasting gains in intercultural sensitivity and L2 motivation. Willingness to communicate, however, plateaued after its initial improvement. This suggests that virtual practice may still require continued real-world reinforcement. One possible explanation for the difference between AR and VR is the way cultural content related to learner activity. AR directly linked cultural meaning to visible objects and locations that learners examined. VR created broader settings in which cultural encounters could be experienced. However, the learning value of these settings depended on clear cultural goals, guidance, and reflection. The lower VR estimate may therefore reflect differences in task alignment, instructional structure, and technical or cognitive demands. It may not be caused by immersion itself. This interpretation remains tentative because the AR estimate was based on only two independent studies. The proposed mechanisms were also not directly tested. The observed difference may therefore reflect differences in tasks, instructional designs, and outcome measures across the included studies. It may not reflect AR or VR alone. Comparator conditions may also have contributed to the observed differences. Gao et al. (2021), Knutzen et al. (2025), and B. Zhang et al. (2022) used technology-based comparators, whereas most AI and AR studies used conventional instruction. These differences may partly explain the lower VR estimate. More direct comparisons between AR and VR are needed.

### 4.3. Effects of AI on Intercultural Learning

AI-supported interventions showed a significant positive effect on intercultural learning outcomes. However, the AI interventions differed considerably in their functions, including chatbots, generative AI, intelligent agents, and adaptive systems. Therefore, the pooled AI effect should be interpreted as an average across different AI applications rather than as a single uniform AI effect. Across the included studies, AI was mainly used to support communication, feedback, adaptation, and cultural reflection. J. Liu (2025) used an integrated adaptive system to detect culturally inappropriate expressions, explain the source of the mismatch, and provide more appropriate alternatives. This allowed learners to receive immediate feedback when cultural misunderstandings occurred. L. Sun (2025) used AI mainly for translation and language feedback, which reduced linguistic barriers and supported cultural exchange between Chinese and Malaysian students. Chen and Pan (2026) used an AI agent to respond to learners during pragmatic conflicts and to provide more guidance after unsuccessful attempts. The clearest improvement appeared in critical cultural awareness rather than intercultural sensitivity or critical reflection. J. Sun and Nie (2026) used culturally biased AI responses as materials for comparison and critique, and the largest effect appeared in metacognitive CQ rather than factual cultural knowledge. Taken together, these studies suggest that AI may support intercultural learning by making cultural misunderstandings, alternative interpretations, and biased assumptions more visible during the learning process. Its contribution may therefore go beyond personalization and include immediate explanation, correction, comparison, and reflection. However, these effects were often supported by teacher-designed tasks, structured reflection, bias-checking activities, and interaction with human partners. This suggests that the effectiveness of AI may depend not only on the technology itself, but also on how its functions were integrated into intercultural learning tasks.

### 4.4. Emerging Technology Supported Cognitive, Attitudinal, and Behavioral Outcomes

Outcome domain did not significantly moderate the effects of emerging technology-supported intercultural learning. This finding indicates that no reliable differences in effect size emerged among cognitive, attitudinal, and behavioral outcomes. It should not be interpreted as evidence that the three domains are identical. More precisely, none of the outcome domains consistently showed a larger technology-supported effect across the included studies. Cognitive, attitudinal, and behavioral outcomes still represent distinct aspects of intercultural learning. However, emerging technologies may support these aspects through different learning activities, making their effects less likely to be consistently concentrated in a single domain.

The included studies show that the three outcome domains represent different learning functions. Cognitive outcomes mainly reflect whether learners can recognize and interpret cultural meanings. From a cognitive perspective, this involves acquiring and understanding cultural knowledge, processing culturally situated information, taking different perspectives, and adapting or revising prior interpretations in response to new cultural cues and feedback (Al-Afifi et al., 2026). Andayani et al. (2026) used AR marker cards to present three-dimensional cultural objects, animations, and audio explanations. Learners were required to connect the visible objects with their corresponding cultural symbols, and the most pronounced improvement occurred in cultural-symbol recognition. Attitudinal outcomes reflect how learners perceive cultural differences and reconsider their own cultural positions. W. Wu and Sun (2025) organized AI-supported role-playing activities around cultural stereotypes, nonverbal communication, cultural conflict, and cultural adaptation. They then examined changes in learners’ intercultural orientations. Behavioral outcomes concern whether learners can act appropriately during intercultural communication. Lv and Chen (2026) required learners to adjust the formality of their expressions, coordinate verbal and nonverbal cues, and make pragmatic repairs when misunderstandings occurred during culturally situated role-plays. These studies suggest that the three domains do not simply measure the same ability. Instead, they capture cultural understanding, cultural orientation, and communicative performance, respectively. These functions also show conceptual links with CQ. Cognitive and behavioral outcomes relate to corresponding CQ dimensions, whereas attitudinal outcomes are distinct from motivational CQ, which concerns intercultural motivation and confidence (Ang et al., 2007). Metacognitive CQ is also relevant because it involves reflecting on and revising cultural interpretations when unfamiliar situations are encountered. These links are theoretical and were not directly tested in the present three-level meta-analysis. Willingness to communicate was coded as Behavioral because it reflects behavioral intention, although it overlaps with motivational CQ. J. Sun and Nie (2026) reported the largest improvement in metacognitive CQ, whereas Knutzen et al. (2025) found no significant CQ advantage for social VR.

However, distinguishing the three outcome domains conceptually and functionally does not, by itself, explain why their effect sizes did not differ significantly. One possible explanation is that many of the included studies did not confine technology-supported learning activities to a single domain. Fathi et al. (2025) placed learners in VR scenarios involving markets, restaurants, and travel. Through the same set of activities, learners acquired cultural knowledge, recognized cultural diversity, developed a willingness to communicate, and practiced communication skills. L. Sun (2025) showed that AI-supported COIL project similarly addressed multiple outcome domains. AI provided translation support and language feedback, while Chinese and Malaysian students developed cultural understanding, intercultural attitudes, and communication skills through collaborative tasks. J. Sun and Nie (2026) further designed AI-supported cultural dialogues that progressed from cultural knowledge and social norms to the management of misunderstandings, identity negotiation, and critical reflection on cultural bias. The same intervention therefore engaged both CQ and intercultural sensitivity. Jiang et al. (2026) showed that VR-based workplace conflict activities also influenced behavioral openness, interpersonal communication, and cultural self-evaluation. Declines in some self-evaluations may reflect learners’ growing awareness of the complexity of cultural conflict rather than poorer learning outcomes (Jiang et al., 2026). Taken together, these interventions did more than address multiple outcome domains in parallel. They connected cultural understanding, attitudinal adjustment, and communicative action within the same learning process. Learners’ understanding of cultural information may shape their cultural judgments and communicative responses, while communicative practice and feedback may prompt them to reinterpret that information and further adjust their attitudes and subsequent behavior. Cognitive, attitudinal, and behavioral outcomes may interact during the learning process. This cyclical, cross-domain process provides one possible interpretation of why outcome domain did not emerge as a significant moderator.

This pattern may reflect a shared process through which emerging technologies support intercultural learning. Learners first encounter a specific cultural situation or difference. They then interpret its cultural meaning, reconsider their initial judgments, and attempt to respond appropriately during communication. Feedback from the task may subsequently lead them to revise their previous understanding. Different technologies may support different stages of this process, while individual studies may measure different outcomes arising from it. Thus, although cognitive, attitudinal, and behavioral outcomes are conceptually distinct, they may be jointly supported by the same learning process in technology-based interventions. The nonsignificant moderation effect is therefore better understood as indicating that the current evidence does not identify any intercultural learning domain as consistently more responsive to emerging technologies. It does not demonstrate that the three domains are indistinguishable. Because the present moderator analysis did not directly test this developmental sequence or any mediating relationships among the domains, the proposed recursive relationship should be regarded as a hypothesis for future research rather than an empirically established mechanism.

### 4.5. Emerging Technology Supported Intercultural Learning Across Academic Levels

Academic level emerged as a significant moderator in the univariable analysis. However, this finding should be regarded as exploratory. The K–12 category contained only two independent studies, both of which were large-sample studies and represented AI and AR interventions. Academic level therefore overlapped with technology type and sample size in the available evidence base, and the observed difference cannot be interpreted as an independent effect of educational stage.

The K–12 studies mainly addressed relatively foundational intercultural learning needs. Andayani et al. (2026) illustrate this pattern. AR cards represented cultural traditions through 3D objects, animations, and audio narration, turning cultural information that might otherwise remain abstract into something that younger learners could directly observe and explore. J. Liu (2025) similarly used an AI-enhanced system that provided personalized learning paths, real-time feedback, voice interaction, and cultural context simulation. These features organized cultural information into a relatively guided learning process. Across these studies, technology did not simply increase exposure to cultural content. It helped structure what learners attended to, made cultural information easier to interpret, and provided support while learners were developing initial connections between language, culture, and social context. This suggests that emerging technologies may be particularly useful at earlier stages of intercultural learning when they make unfamiliar cultural information concrete and provide explicit scaffolding for its interpretation.

The adult studies reflected a different set of demands. Adult learners were more often required to use cultural understanding in communication, collaboration, professional practice, judgment, and reflection. In L. Sun (2025), Chinese and Malaysian university students participated in AI-supported COIL activities in which they had to explain their ideas, respond to different perspectives, and negotiate cultural meanings during collaborative work. Technology facilitated the interaction, but intercultural learning depended on what learners did with the perspectives they encountered. Chen and Pan (2026) showed a similar pattern through AI-agent simulations. Learners were placed in intercultural situations in which they had to judge culturally appropriate responses, participate in role play, and reflect on their decisions. In these settings, technology was no longer primarily a means of presenting cultural information. Instead, it created situations in which existing knowledge had to be applied, questioned, and revised. This distinction is important because adult learners generally enter intercultural learning with more established experiences, communication habits, and interpretive frameworks. Their learning needs may therefore involve not only acquiring new cultural information but also reconsidering existing assumptions and learning how to act when different cultural perspectives come into contact.

Taken together, these cross-study patterns suggest, but do not establish, a possible developmental alignment between learning needs and technological support across academic levels. At earlier stages, intercultural learning may place greater emphasis on constructing basic cultural representations and recognizing meaningful differences. Technologies that visualize cultural content, organize information, and provide immediate guidance may support these processes. At later stages, learning tasks may involve more application of cultural understanding in uncertain and interactive situations, where technology can provide opportunities for perspective comparison, meaning negotiation, situated decision making, and reflection. Accordingly, the proposed match between developmental learning needs and technological support should be regarded as an exploratory hypothesis rather than a mechanism established by the moderator analysis. Further studies with a more balanced distribution of technologies, academic levels, and sample sizes are needed to examine this possibility directly.

### 4.6. Emerging Technology Supported Intercultural Learning Across Sample Sizes

Sample size was a significant moderator, with larger-sample studies reporting stronger effects. This pattern contrasts with the small-study effects reported in some previous educational research (Cheung & Slavin, 2016; Slavin & Smith, 2009). However, because sample size overlapped with technology type and academic level, the observed association cannot be attributed to sample size alone.

One possible reason for this pattern may relate to how technology supports learning in larger groups. In conventional large classes, teachers may have limited opportunities to interact continuously with every learner and provide timely feedback (Schaffer et al., 2017; L. Wang & Calvano, 2022). However, intercultural learning often requires learners to interpret cultural situations repeatedly, test different communication strategies, and revise their understanding and behavior through reflection and feedback. Emerging technologies may support teachers by undertaking part of the task delivery, practice organization, and feedback process. At the same time, technology-supported feedback and guided reflection may reduce some of the instructional demands placed on teachers. These functions may therefore help teachers provide relatively consistent learning support to a larger group.

Several large-sample studies provide examples of how emerging technologies may be used to support instruction in relatively large groups. W. Wu and Sun (2025) used a simulation platform to deliver an eight-week sequence of intercultural tasks to 307 students. Each student participated in weekly activities that followed a consistent process of briefing, live simulation, debriefing, and feedback. It also enabled students to receive comparable opportunities for participation and reflection. J. Liu (2025) used an AI system with 262 students. The system organized learning paths and cultural simulations while also providing speech support and real-time feedback. As a result, individual students could receive timely guidance within a large group. Andayani et al. (2026) implemented ten AR sessions with 100 elementary students from four schools. Standardized AR materials gave each group access to the same cultural tasks, while teachers guided students’ exploration and discussion in smaller groups. This arrangement may have allowed teachers to focus more on guidance and reflection while the technology supported the delivery of learning content. Together, these studies suggest that emerging technologies may help teachers manage large-scale instruction by distributing structured tasks, supporting repeated practice, and making feedback opportunities available to more learners. Although the studies do not show that every student received fully individualized support, they indicate that technology may help teachers provide a basic and relatively consistent level of support across a large group.

Therefore, the stronger effects reported in some large-sample studies should not be attributed directly to the number of participants. Sample size was classified using operational cutoffs, and the large-sample category also overlapped with technology type and academic level. Given the limited number of independent studies, the observed pattern may partly reflect these study characteristics or the way in which sample size was categorized. Sample size should therefore be treated as a study characteristic associated with variation in effect estimates rather than as a causal mechanism affecting intervention effectiveness. Given the limited number of independent studies (n = 17), sample size and technology type were not simultaneously included in a multivariable model. Therefore, the observed sample-size moderation cannot be interpreted as an effect independent of technology type. The moderator analysis examined sample size rather than scalability itself. The possibility that emerging technologies may help organize tasks, practice, and feedback in larger groups remains a plausible interpretation, but it should be regarded as an exploratory hypothesis for future research rather than a mechanism established by the present moderator analysis.

### 4.7. Emerging Technology Supported Intercultural Learning Across Measurement Instrument Types

Measurement instrument type did not significantly moderate the effect sizes in the present study. However, this finding appears to differ from X. Zhang and Zhou (2023), who reported a significant moderating effect. Specifically, their results showed that self-developed tests had the largest effect (r = 0.66), while self-developed scales (r = 0.38) and validated scales (r = 0.33) showed smaller and more similar effects. This suggests that the significant result reported by X. Zhang and Zhou (2023) may have been mainly related to self-developed tests. One possible explanation is that these tests are often closely linked to the specific knowledge or skills taught in an intervention. Therefore, they may be more sensitive to intervention-related changes. In contrast, broader scales usually assess more general intercultural attitudes or competence and may be less directly linked to the intervention content. In the present study, a broader classification was used. Study-specific and substantially modified instruments were grouped as Adapted and compared with Established measures. This grouping did not distinguish self-developed tests from self-developed scales. Therefore, the particularly large effect of self-developed tests may have been reduced when different adapted instruments were combined within one category. In this three-level meta-analysis, adapted measures did not produce significantly larger effects than Established measures. Taken together, these findings suggest that measurement effects may depend more on the type of instrument and its alignment with the intervention than on whether the measure is adapted or established. They also suggest that the relatively large overall effect in the present meta-analysis cannot be explained simply using adapted measurement instruments.

## 5. Implications

### 5.1. Technology Use and Intercultural Learning Outcomes

The first practical implication is that technology selection should be guided by instructional tasks and the support they require. AR and VR primarily provide learning environments and ways of presenting cultural contexts, whereas AI provides functions such as language support, explanation, and feedback. They should therefore not be treated as equivalent or mutually exclusive technology options. Teachers can first select a learning environment according to the intercultural task. AR may be useful when learners need to interpret cultural meanings embedded in physical objects, symbols, or places. VR may be appropriate when learners need to apply cultural understanding in simulated social or professional situations. If learners also require language assistance, cultural explanations, or feedback on their responses, AI could potentially be integrated into AR- or VR-based environments to provide additional support. This proposed integration draws on their complementary functions, but its effectiveness requires direct empirical testing.

The nonsignificant differences among cognitive, attitudinal, and behavioral outcomes also suggest that technology-supported activities do not need to be limited to a single learning domain. Intercultural learning activities can connect cultural understanding, reflection, and communicative behavior within the same process. Learners may first identify and interpret a cultural phenomenon, then reconsider their initial judgments, and subsequently attempt to respond in interaction. Different technological functions can support different parts of this process rather than being tied separately to cognitive, attitudinal, or behavioral outcomes. This sequence should be understood as a design interpretation rather than a developmental process established by the meta-analysis. The practical focus can therefore shift from identifying the best technology for a particular outcome to selecting appropriate technological functions according to the learning objective and using suitable instructional design so that these functions jointly support a coherent intercultural learning process.

### 5.2. Implications for Different Academic Levels

The second practical implication is that different learner groups may require different forms and levels of technological support rather than fundamentally different technologies. For K–12 learners, technology can be used more to make unfamiliar cultural meanings concrete and to provide a clearer structure for interpretation. Learning activities can begin with concrete cultural objects, practices, or social situations and then guide learners to identify their meanings, compare them with familiar cultural examples, and explain relevant differences. Visual representations, brief explanations, and immediate feedback can support this process, while teachers can provide necessary cultural context and help learners avoid oversimplified or stereotypical interpretations. Technology design for K–12 education may therefore place greater emphasis on the accessibility of cultural content and the instructional scaffolding needed during learning. It is worth noting that the implications for K–12 learners should remain tentative because this category was represented by only two independent studies and 12 effect sizes.

For adult learners, technology can be used to help them apply cultural understanding in communication and decision-making. Learning activities can ask learners to respond to culturally different viewpoints, explain their choices, or deal with misunderstandings in intercultural situations. AI, VR, and AR can provide feedback, alternative perspectives, or simulated situations that encourage learners to reconsider their initial responses. This may be particularly useful in higher education and professional learning, where learners often need to communicate and collaborate with people from different cultural backgrounds (Guillén-Yparrea & Ramírez-Montoya, 2023). Therefore, technology-supported learning for adults may place greater emphasis on using and revising cultural understanding through interaction and practice. However, these implications should be interpreted cautiously because academic level overlapped with technology type and sample size in the included studies.

### 5.3. Implications for Large-Scale Implementation

The third practical implication is that, in larger-scale instruction, the value of emerging technologies may extend beyond small-scale learning to the organization and maintenance of core learning opportunities. Standardized task sequences, repeated practice opportunities, and technology-supported feedback can allow more learners to participate in comparable intercultural learning activities. Technology may also take on part of the routine work involved in task delivery, practice organization, and feedback, which may allow teachers to devote more attention to cultural explanation, classroom discussion, and reflection.

From this perspective, the role of technology in larger-scale intercultural learning can be understood as a redistribution of instructional work rather than a replacement of teachers. Technology can help learners maintain access to core tasks, practice, and basic feedback, while teachers can focus more on interpreting cultural meanings, addressing stereotypes, and guiding discussion in complex or ambiguous situations. Such a division of instructional functions may help maintain relatively stable learning support as the scale of instruction increases. However, this interpretation should be treated cautiously because sample size overlapped with technology type and academic level in the included studies. The potential role of technology in supporting task organization, practice, and feedback at scale should therefore be regarded as a possible practical implication rather than a mechanism directly established by the moderator analysis.

## 6. Limitations

Several limitations should be considered. First, although 77 effect sizes were included, they came from only 17 independent studies, and some subgroups, particularly AR and K–12, were represented by only two studies. Given the limited number of independent studies, the moderator analyses had limited statistical power and should therefore be interpreted as exploratory. Technology type, academic level, and sample size also overlapped, so subgroup and moderator findings should be interpreted cautiously. Although the three-level model accommodated the hierarchical structure of multiple effect sizes, correlated sampling errors were not explicitly modeled in the primary analysis because the necessary outcome correlations were rarely reported. The sensitivity analyses indicated that the overall pooled effect was stable across assumed within-study correlations from ρ = 0.30 to 0.70. However, these analyses did not assess the stability of moderator findings. Furthermore, cluster-robust inference was not conducted, and the reported moderator tests therefore remain subject to uncertainty associated with the limited number of independent studies and potentially correlated sampling errors. Second, the risk-of-bias profile also limits confidence in the precise magnitude of the pooled effect. All randomized studies had some concerns, while most non-randomized studies had moderate risk and two had serious risk. Although excluding the two serious-risk studies did not materially change the overall result, methodological concerns remained in the rest of the evidence base. Although comparator conditions were descriptively classified, their potential moderating effects were not examined. Therefore, their contribution to heterogeneity and differences between technology types remains uncertain. Third, substantial heterogeneity remained. This may be due to differences in learning tasks, intervention duration, learner characteristics, measurement tools, and cultural contexts, which were not consistently reported across studies. Furthermore, novelty and expectancy effects could not be ruled out, particularly because several interventions were relatively short and most studies used conventional instruction as the comparison condition. Fourth, the generalizability of the findings should be interpreted cautiously because the review included only English-language studies and because learner populations, technologies, and cultural contexts were unevenly represented. The pooled estimates may therefore not generalize equally to other linguistic, educational, or cultural settings. Fifth, the review relied mainly on reported outcome data and could not examine learning processes in detail. Sixth, the protocol was not prospectively registered. Finally, the proposed mechanisms were inferred rather than directly tested, and AI, AR, and VR were coded as broad technology categories. Future research should examine these mechanisms and more specific technological functions.

## 7. Conclusions

This three-level meta-analysis found a strong positive effect of emerging technology-supported intercultural learning. However, the risk-of-bias profile of the included studies suggests that greater confidence can be placed in the positive direction of the effect than in its precise magnitude. The included interventions provided different ways of engaging learners with cultural content, although the contribution of specific instructional features and novelty effects could not be isolated. AR, AI, and VR appeared to support different relationships between cultural information and learner activity, while cognitive, attitudinal, and behavioral outcomes may develop through an interconnected learning process rather than in isolation. Differences across academic levels and sample-size groups should be interpreted more cautiously because these characteristics overlapped with technology type in the available evidence base. Overall, the findings shift attention from whether emerging technologies work to how their functions, learning tasks, and instructional designs interact to support intercultural learning.

## Figures and Tables

**Table 1:** Comparison of Previous Quantitative Syntheses and the Present Study.

Study	Period	Technologies	Outcome Classification	Model	Moderators	Overlap with Present Review
X. Zhang and Zhou (2023)	2000–2021	Computer simulation; blended learning; digital materials/tools; computer-mediated communication	Overall intercultural competence; affective, cognitive, behavioral	Conventional meta-analysis (r; CMA 2.0)	Intervention type; instrument type; grade level	0/17
Shadiev et al. (2026)	2017–2025	Online course systems; multimedia; social media; VR/AR; online meeting tools; AI language tools	Intercultural competence and related measured constructs	Random-effects analyses by design; within-study aggregation (CMA 3.3)	Instrument; academic level; technology; intervention type; duration; publication year; sample size	Not verifiable
Xiao et al. (2026)	2020–2025	Online communication; immersive experiences	Knowledge; skills; attitudes	Three-level random-effects meta-analysis (REML; metafor)	Course type; intervention type; duration; cultural background	3/17
Present study	2020–2026	AI, AR and VR	Cognitive; attitudinal; behavioral	Three-level random-effects meta-analysis (metafor)	Technology; outcome domain; academic level; sample size; measurement instrument	

Note. AI = artificial intelligence; AR = augmented reality; VR = virtual reality; CMA = Comprehensive Meta-Analysis; REML = restricted maximum likelihood. Overlap refers to the number of primary studies shared with the present review (N = 17). The overlap with Shadiev et al. (2026) could not be verified because the complete list of included studies was unavailable in the main article.

**Table 2:** Characteristics of Included Studies (N = 17).

Authors (Year)	Year	N_E/N_C	Technology Type	Outcome Type	Learner Level	Sample Size	Measurement Instrument Type	Intervention Duration
Gao et al. (2021)	2021	25/25	VR	Cognitive; Behavioral	Adults	Medium	Adapted	≤4 weeks
B. Zhang et al. (2022)	2022	4/4	VR	Cognitive; Attitudinal; Behavioral	Adults	Small	Adapted	≤4 weeks
S. Liu et al. (2023)	2023	25/23	AR	Attitudinal; Behavioral	Adults	Small	Established	>8 weeks
Fathi et al. (2025)	2025	25/25	VR	Cognitive; Attitudinal; Behavioral	Adults	Medium	Established	>8 weeks
L. Sun (2025)	2025	38/39	AI	Cognitive; Attitudinal; Behavioral	Adults	Medium	Established	>8 weeks
J. Liu (2025)	2025	131/131	AI	Cognitive; Attitudinal; Behavioral	K–12	Large	Adapted	>8 weeks
W. Wu and Sun (2025)	2025	153/154	AI	Cognitive; Behavioral	Adults	Large	Established	5–8 weeks
Risda et al. (2025)	2025	126/126	AI	Cognitive; Attitudinal	Adults	Large	Adapted	5–8 weeks
Knutzen et al. (2025)	2025	14/13; 10/11	VR	Cognitive	Adults	Small	Adapted	Unclear
Chen and Pan (2026)	2026	20/22	AI	Cognitive; Behavioral	Adults	Small	Established	>8 weeks
J. Sun and Nie (2026)	2026	32/30	AI	Cognitive; Attitudinal; Behavioral	Adults	Medium	Established	5–8 weeks
Z. Wang (2026)	2026	30/30	AI	Cognitive; Attitudinal; Behavioral	Adults	Medium	Unclear	Unclear
Y. Wang et al. (2026)	2026	23/25	VR	Attitudinal; Behavioral	Adults	Small	Established; Adapted	5–8 weeks
Lv and Chen (2026)	2026	32/32	AI	Cognitive; Behavioral	Adults	Medium	Adapted	≤4 weeks
Mahmoudi-Dehaki and Nasr-Esfahani (2026)	2026	71/71	AI	Cognitive; Behavioral	Adults	Large	Established	>8 weeks
Andayani et al. (2026)	2026	50/50	AR	Cognitive; Attitudinal; Behavioral	K–12	Large	Established; Adapted	5–8 weeks
Jiang et al. (2026)	2026	174/26	VR	Attitudinal; Behavioral	Adults	Large	Established; Adapted	Unclear

Note. N_E/N_C refers to the sample size of the experimental group and control group. For Knutzen et al. (2025), the different sample sizes reflect outcome-specific response availability within the same participant groups.

**Table 3:** Coding of Intercultural Learning Outcomes.

Domain	Definition and Included Outcomes	Boundary Treatment
Cognitive	Cultural knowledge, literacy, understanding, cognitive CQ, and knowledge-oriented cultural awareness.	Cultural awareness was coded as Cognitive when it assessed recognition, knowledge, or understanding; it was coded as Attitudinal when it assessed evaluation, openness, or acceptance. Inseparable multidimensional measures were excluded from domain-specific analyses.
Attitudinal	Intercultural sensitivity, openness, respect, empathy, and motivation explicitly directed toward intercultural engagement.	General learning motivation and engagement were excluded. Anxiety was not coded as Attitudinal because it represents an affective state rather than an evaluative disposition toward cultural differences.
Behavioral	Intercultural communication skills, adaptation, communication strategies, behavioral intentions, and culturally appropriate performance.	Willingness to communicate was coded as a behavioral intention. Speaking and pragmatic performance were included only when their scoring criteria explicitly assessed intercultural interaction or cultural appropriateness rather than general language proficiency. Engagement was included only when it represented observable participation in intercultural interaction.

**Table 4:** Moderator analysis comparing AR- and VR-based interventions.

Moderator Variables	N	K	Estimate (*β*)	SE	95% CI	*t*	*p*	*QE* (df)
AR/VR								186.360 (32) ***
Intercept: AR (ref.)	2	10	1.856	0.509	[0.820, 2.893]	3.648	<.001	
VR	6	24	−1.481	0.589	[−2.681, −0.280]	−2.513	.017	

Note. AR was used as the reference group. The intercept represents the estimated effect size for AR-based interventions. The coefficient for VR represents the estimated difference between VR- and AR-based interventions. *** *p* < .001. N = number of independent studies; K = number of effect sizes.

**Table 5:** Overall effect of AI-supported intercultural learning.

Intervention	N	K	Estimate (g)	SE	95% CI	*t*	*p*	*Q* (df)
AI	9	43	1.150	0.201	[0.744, 1.555]	5.718	<.001	537.709 (42) ***

Note. AI-supported interventions were analyzed separately from AR- and VR-based interventions. The analysis included 43 effect sizes from nine independent studies. *Q* indicates heterogeneity among effect sizes. *** *p* < .001. N = number of independent studies; K = number of effect sizes.

**Table 6:** Moderator analysis by outcome domain.

Moderator Variables	N	K	Estimate (*β*)	SE	95% CI	*t*	*p*	*QE* (df)
Outcome domain								1108.567 (74) ***
Intercept: Cognitive (ref.)	14	27	0.892	0.212	[0.469, 1.315]	4.206	<.001	
Attitudinal	11	27	−0.028	0.155	[−0.337, 0.281]	−0.180	.858	
Behavioral	15	23	0.239	0.148	[−0.056, 0.533]	1.616	.110	

Note. Cognitive outcomes were used as the reference group. The intercept represents the estimated effect size for cognitive outcomes. The coefficients for attitudinal and behavioral outcomes represent their estimated differences from the cognitive outcome group. *QE* indicates residual heterogeneity. *** *p* < .001. N = number of independent studies; K = number of effect sizes.

**Table 7:** Moderator analysis by academic level.

Moderator Variables	N	K	Estimate (*β*)	SE	95% CI	*t*	*p*	*QE* (df)
Academic level								689.309 (75) ***
Intercept: Adult learners (ref.)	15	65	0.758	0.145	[0.469, 1.047]	5.228	<.001	
K–12 learners	2	12	1.679	0.404	[0.874, 2.484]	4.156	<.001	

Note. Adult learners were used as the reference group. The intercept represents the estimated effect size for adult learners. The coefficient for K–12 learners represents the estimated difference between K–12 learners and adult learners. *QE* indicates residual heterogeneity. *** *p* < .001. N = number of independent studies; K = number of effect sizes.

**Table 8:** Moderator analysis by sample size.

Moderator Variables	N	K	Estimate (*β*)	SE	95% CI	t	*p*	*QE* (df)
Sample size								861.335 (74) ***
Intercept: Large sample size (ref.)	6	30	1.575	0.274	[1.029, 2.121]	5.748	<.001	
Medium sample size (50–99)	6	32	−0.958	0.387	[−1.730, −0.186]	−2.472	.016	
Small sample size (<50)	5	15	−0.960	0.423	[−1.802, −0.117]	−2.269	.026	

Note. Large sample size was used as the reference group. The intercept represents the estimated effect size for studies with large samples. The coefficients for medium and small samples represent the estimated differences from the large-sample group. *QE* indicates residual heterogeneity. *** *p* < .001. N = number of independent studies; K = number of effect sizes.

**Table 9:** Moderator analysis by measurement instrument type.

Moderator Variables	N	K	Estimate (*β*)	SE	95% CI	*t*	*p*	*QE* (df)
Measurement instrument type								975.226 (72) ***
Intercept: Established (ref.)	10	36	0.858	0.230	[0.399, 1.317]	3.722	<.001	
Adapted	9	38	0.296	0.238	[−0.178, 0.770]	1.244	.218	

Note. Established measures were used as the reference group. The intercept represents the estimated effect size for established measures. The coefficient for adapted measures represents the estimated difference between adapted and established measurement instrument type. *QE* indicates residual heterogeneity. *** *p* < .001. N = number of independent studies; K = number of effect sizes.

## Data Availability

The data analyzed in this meta-analysis were extracted from previously published studies cited in the article. The extracted data used for the meta-analysis and the analytic code are available from the corresponding author upon reasonable request.

## Supplementary Materials

The following supporting information can be downloaded at: https://www.mdpi.com/article/10.3390/jintelligence14100265/s1, PRISMA 2020 checklist.

## Appendix A. The Selected Articles from the Mainstream Databases

**Database**	**Exact Search Strategy**	**Records Retrieved (n)**	**Final Search Date**
Web of Science	(“intercultural learning” OR “intercultural education” OR “intercultural competence” OR “intercultural communicate” OR “intercultural awareness” OR “intercultural sensitivity” OR “intercultural skill” OR “cross-cultural learning” OR “cross-cultural education” OR “cross-cultural competence” OR “cross-cultural communicate” OR “cross-cultural skill” OR “cultural competence” OR “cultural awareness” OR “cultural intelligence” OR “global competence”) (All Fields) and (“artificial intelligence” OR “generative AI” OR “AI-supported” OR “AI-assisted” OR “AI-enhanced” OR ChatGPT OR chatbot OR “conversational agent” OR “intelligent tutoring system” OR “intelligent language learning system” OR “large language model” OR “augmented reality” OR “virtual reality” OR “extended reality” OR “mixed reality” OR “immersive technology” OR “immersive virtual environment” OR “virtual world” OR “metaverse”) (All Fields)	668	31 July 2026
Scopus	TITLE-ABS-KEY ((“intercultural learning” OR “intercultural education” OR “intercultural competence” OR “intercultural communicative competence” OR “intercultural communication competence” OR “intercultural awareness” OR “intercultural sensitivity” OR “intercultural skill” OR “cross-cultural learning” OR “cross-cultural education” OR “cross-cultural competence” OR “cross-cultural communication competence” OR “cross-cultural skill”) AND (“artificial intelligence” OR “generative AI” OR “AI-supported” OR “AI-assisted” OR “AI-enhanced” OR ChatGPT OR chatbot OR “conversational agent” OR “intelligent tutoring system” OR “intelligent language learning system” OR “large language model” OR “augmented reality” OR “virtual reality”) AND (learn OR educate OR teach OR student OR learner OR classroom OR course OR train OR intervention))	350	31 July 2026
ProQuest	TIABSU ((“intercultural learning” OR “intercultural education” OR “intercultural competence” OR “intercultural communicative competence” OR “intercultural communication competence” OR “intercultural awareness” OR “intercultural sensitivity” OR “cross-cultural learning” OR “cross-cultural education” OR “cross-cultural competence” OR “cross-cultural communicative competence” OR “cross-cultural communication competence”) AND (“artificial intelligence” OR “generative artificial intelligence” OR “generative AI” OR “AI-supported” OR “AI-assisted” OR “AI-enhanced” OR ChatGPT OR chatbot OR “conversational agent” OR “intelligent tutoring system” OR “intelligent language learning system” OR “large language model” OR “augmented reality” OR “virtual reality”))	140	31 July 2026

## Appendix B. General Overview of Selected Studies (N = 17)

**Research Purpose**	**Study Results and Observations**	**References**	**Comparator Type**
To examine whether a VR-based Christmas culture learning environment improves cultural knowledge, behavioral performance, and intercultural sensitivity among Chinese EFL learners compared with a non-VR condition.	The study found no significant advantage of VR over the non-VR method across knowledge, behavior, and attitude outcomes. However, most learners preferred the VR method, and presence was positively associated with intercultural interaction engagement.	Gao et al. (2021)	Technology-based
To investigate whether 3D contextual interactive games in a gamified virtual environment can support intercultural collaboration and cultural learning among Chinese and Irish students.	The virtual environment provided shared rooms, avatars, contextual tasks, and cultural artifacts. The findings indicated that the 3D interactive games supported collaborative cultural learning more effectively than text-based assignments.	B. Zhang et al. (2022)	Technology-based
To examine the effect of AR-based language instruction on intercultural competence and L2 learning motivation among Chinese EFL learners.	Learners who received AR-based instruction showed significantly higher intercultural competence and L2 learning motivation than those in traditional instruction. Qualitative findings suggested that AR increased engagement and deepened cultural understanding.	S. Liu et al. (2023)	Conventional/non-technology-centred
To evaluate whether a VR application can improve EFL learners’ intercultural communicative competence and willingness to communicate.	The VR-enhanced group showed significant gains in intercultural communicative competence and willingness to communicate. Interviews suggested benefits in confidence, cultural awareness, language practice, and real-world applicability, but also noted technical and interactional limitations.	Fathi et al. (2025)	Conventional/non-technology-centred
To investigate the impact of an AI-enabled Collaborative Online International Learning program on the intercultural competence of Chinese English majors.	The AI-enabled COIL program produced significant improvements in intercultural competence, especially in attitudes, intercultural communication skills, and intercultural consciousness. Qualitative findings showed that AI tools and collaboration supported cross-cultural understanding.	L. Sun (2025)	Conventional/non-technology-centred
To evaluate whether AI-personalized, niche-specific English for Tourism materials developed within a human-in-the-loop framework can improve tourism students’ English proficiency and intercultural communicative competence.	The experimental group showed significantly greater improvements in English proficiency and intercultural communicative competence than the control group. Qualitative findings indicated increased engagement and emphasized the importance of instructor mediation in correcting AI inaccuracies, addressing cultural and ethical issues, and preventing excessive reliance on AI feedback.	Mahmoudi-Dehaki and Nasr-Esfahani (2026)	Conventional/non-technology-centred
To examine the effects of Gen-AI and multimedia-based intercultural Japanese language teaching on intercultural communication adaptation, L2 motivation, and Japanese language proficiency.	The Gen-AI and multimedia-based intervention improved intercultural communication adaptation, learning motivation, and Japanese language proficiency. The study suggests that AI-supported multimedia instruction can strengthen cultural and language learning outcomes.	Risda et al. (2025)	Conventional/non-technology-centred
To evaluate whether a social VR seminar can support intercultural communication learning and CQ motivation in distance learning.	The study compared a social VR and videoconferencing condition with a videoconferencing-only condition. The findings indicated that social VR can support intercultural communication learning, but its effectiveness depends on platform design, onboarding, technical access, and task structure.	Knutzen et al. (2025)	Technology-based
To examine the effectiveness of a VR-based cultural immersion program for EFL learners’ intercultural sensitivity, willingness to communicate, and L2 learning motivation over time.	The VR group showed stronger immediate gains than the control group in intercultural sensitivity, willingness to communicate, and L2 motivation. Longer-term results showed sustained growth in intercultural sensitivity and motivation, while willingness to communicate plateaued after initial gains.	Y. Wang et al. (2026)	Conventional/non-technology-centred
To explore how an AI-supported model can promote the transfer of English intercultural communicative competence in higher education.	The study proposed an AI-supported cross-cultural learning resource recommendation model and a concept-map-based intercultural competence cultivation model. The experimental class showed stronger post-intervention performance than the control class on intercultural communicative knowledge and the overall competence score, while some dimensions did not differ significantly.	Z. Wang (2026)	Conventional/non-technology-centred
To examine whether scenario-based AI-agent chat simulations can improve intercultural awareness in an EFL preservice teacher education course.	The AI-supported simulation tasks produced significant gains in overall intercultural competence and critical cultural awareness. The study also provided a structured model with a case library, role-play workflow, adaptive scaffolding, and debriefing feedback.	Chen and Pan (2026)	Conventional/non-technology-centred
To examine whether LLM-mediated voice dialogue can foster intercultural communicative competence among Chinese-as-a-foreign-language learners.	The LLM-dialogue group outperformed the conventional instruction group on intercultural sensitivity and CQ. Metacognitive CQ showed the largest effect, while learners rated cultural learning effectiveness higher than communicative authenticity.	J. Sun and Nie (2026)	Conventional/non-technology-centred
To investigate whether a VR simulation can develop intercultural competence among manufacturing technician students more effectively than lecture-based workplace communication instruction.	The VR group completed immersive workplace simulations and showed stronger gains in behavioral openness and interpersonal communication skills. The lecture group showed no statistically significant changes, suggesting the value of experiential VR training for workforce intercultural competence.	Jiang et al. (2026)	Conventional/non-technology-centred
To compare traditional intercultural pedagogy with simulation-based instruction for developing intercultural competence among international students.	The eight-week AI-based Mursion simulation was compared with traditional instruction for international students. The study reported stronger post-intervention intercultural competence outcomes for the simulation group across several Intercultural Development Inventory dimensions.	W. Wu and Sun (2025)	Conventional/non-technology-centred
To develop and evaluate a multimodal AI framework for cross-cultural pragmatic competence analysis in global English communication.	The MCPAS system combined language modeling, acoustic-prosodic analysis, computer vision, and culture calibration. In a four-week student intervention, the MCPAS-supported group showed larger gains than the conventional teaching group across pragmatic-competence dimensions, with particularly large effects for cultural schema activation and overall pragmatic effectiveness.	Lv and Chen (2026)	Conventional/non-technology-centred
To examine whether AR-based cultural education can enhance cultural awareness, cultural literacy, and student engagement among elementary school students.	The AR group showed significant improvements in cultural awareness, cultural literacy, and engagement. The study also reported that cognitive engagement partly mediated the benefits of AR-supported cultural learning.	Andayani et al. (2026)	Conventional/non-technology-centred
To examine whether an AI-enhanced intelligent language learning system can improve youth learners’ language proficiency and intercultural communication competence.	The AI-enhanced system produced greater improvement than the control condition across language fluency, cultural understanding, communication strategies, and cultural sensitivity. The intervention also showed sustained benefits and emphasized personalized learning paths and real-time feedback.	J. Liu (2025)	Conventional/non-technology-centred
